# Synergistic fungal consortia enhance maize growth and soil biological functions under microplastic stress

**DOI:** 10.1186/s12870-026-08924-w

**Published:** 2026-05-20

**Authors:** Zeeshan Khan, Ghulam Haider, Fazal Adnan, Zeshan Sheikh, Muhammad Faraz Bhatti

**Affiliations:** 1 Department of Agricultural Sciences and Technology, Atta-ur-Rahman School of Applied Biosciences (ASAB), National University of Sciences and Technology (NUST), Islamabad, 44000 Pakistan; 2 Department of Microbiology and Biotechnology, Atta-ur-Rahman School of Applied Biosciences (ASAB), National University of Sciences and Technology (NUST), Islamabad, 44000 Pakistan; 3 Institute of Environmental Sciences and Engineering (IESE), School of Civil and Environmental Engineering (SCEE), National University of Sciences and Technology (NUST), Islamabad, 44000 Pakistan

**Keywords:** Microplastics (MPs), Fungal inoculation, Redox balance, Mycoremidiation, Stress mitigation

## Abstract

**Background:**

Polyethylene terephthalate microplastics (PET-MPs) contamination in agricultural soils is an emerging global concern because these persistent particles disrupt soil structure, nutrient dynamics, and plant physiological processes, ultimately threatening crop productivity and food security. Despite their widespread occurrence in agroecosystem, yet their impacts on crop, soil, and microbe interactions remain poorly understood. Beneficial fungi such as *Trichoderma* and *Metarhizium* are widely recognized for promoting plant growth and stress tolerance, but their combined potential to alleviate microplastic toxicity has not been systematically evaluated. This study investigated how PET-MPs influence maize growth, redox homeostasis, metabolism, and soil biological functioning, and assessed whether single or combined inoculation with *Trichoderma longibrachiatum* and *Metarhizium anisopliae* can mitigate PET − MPs induced stress.

**Results:**

PET − MPs exposure caused strong growth inhibition in maize, with marked decline in biomass, chlorophyll content, and root development, accompanied by enhanced oxidative stress and membrane damage. These physiological injuries were linked to disrupted antioxidant systems, imbalanced redox metabolites, and major reprogramming of root exudates and central carbon metabolism. Soil nutrient availability, enzyme activities, and microbial community structure were also significantly altered. Inoculation with either *Trichoderma* or *Metarhizium* partially mitigated these effects, but their co-application produced a stronger response. The fungal consortium most effectively suppressed reactive oxygen species, restored antioxidant and redox homeostasis, and redirected root metabolism toward sugars, organic acids, and carbon release. At the soil level, the combined fungal inoculation enhanced nitrogen transformations, stimulated key enzymes, and promoted a more active and balanced microbial community under PET-MPs stress.

**Conclusions:**

PET-MPs imposes severe physiological, biochemical, and ecological constraints on maize and its rhizosphere. Synergistic inoculation with *Trichoderma* and *Metarhizium* effectively counteracts these stresses by reinforcing redox balance, metabolic functioning, and soil biological health. This fungal consortium represents a promising, nature − based strategy for improving crop resilience and sustaining soil quality in PET-MPs contaminated agroecosystems.

**Graphical Abstract:**

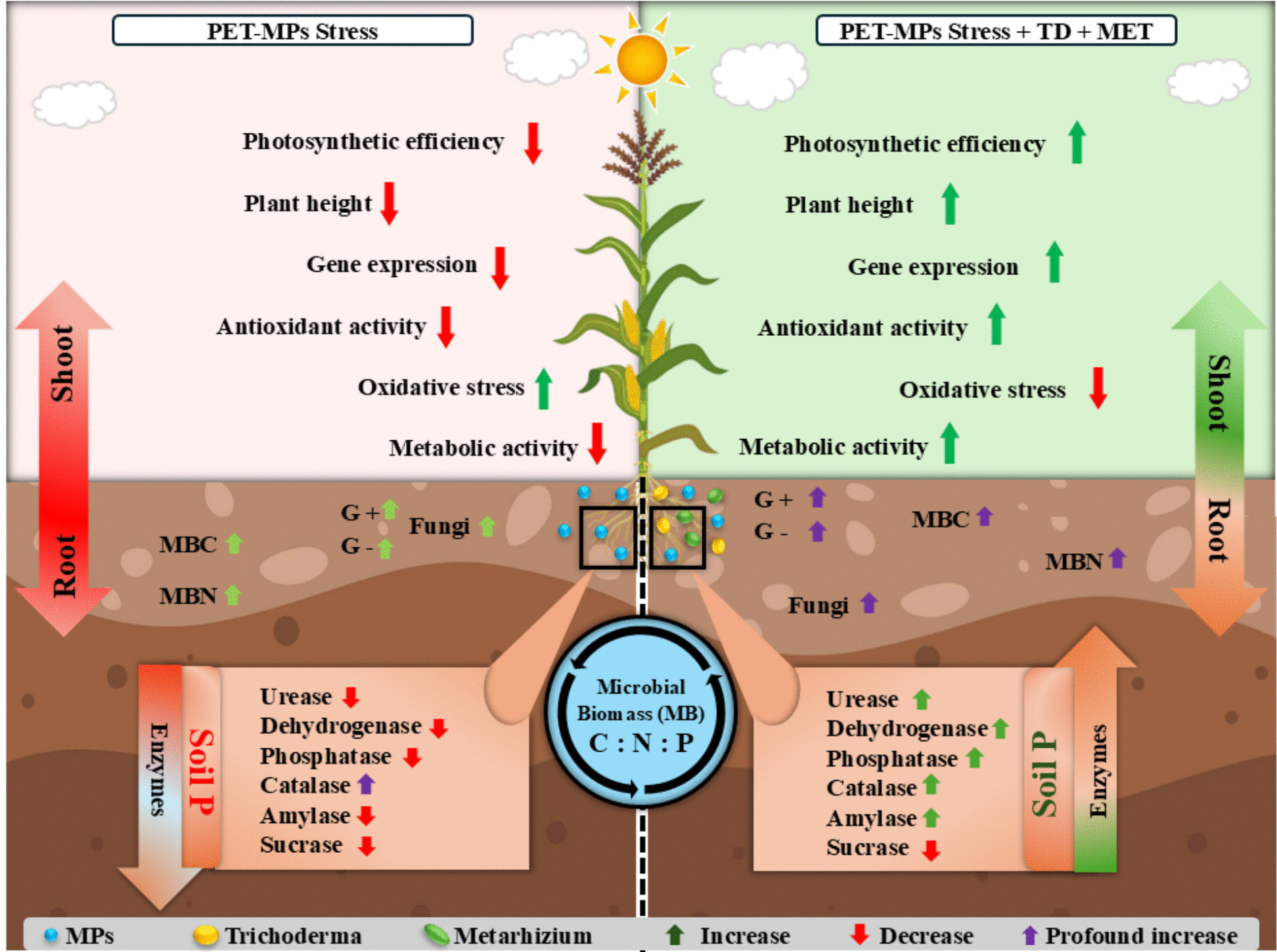

**Supplementary Information:**

The online version contains supplementary material available at 10.1186/s12870-026-08924-w.

## Introduction

Crop plants are continuously subjected to various environmental stress factors that can compromise growth, physiological activity, and productivity [1]. In addition to conventional abiotic stressors, microplastics (MPs) – size < 5 mm, are becoming the new environmental stress factor in agroecosystems. These particles are introduced in soil by plastic mulching, contaminated water irrigation, and plastic waste fragmentation [2–4]. Among different types of MPs, Polyethylene terephthalate (PET) − MPs were selected in this study because they are the most common and ubiquitous MPs in the terrestrial environment because of their widespread use in packaging materials and high environmental resistance [5–8]. The presence of MPs in soil has been associated with adverse effects on growth of plants in agroecosystems as they interfere with their root architecture, limiting nutrient uptake, and redox balance [9]. Furthermore, their accumulation in rhizosphere can modify soil physicochemical characteristics and influence the composition and activity of microbial communities, ultimately affect the soil fertility and plant microbe interaction [10].

Such stress conditions necessitate the exploration of sustainable mitigation strategies, particularly those involving beneficial soil microorganisms. Among these, fungi play a significant role in maintaining soil health and enhancing plant tolerance [11]. Fungal species help in the enhancement of nutrient availability, root growth and stabilization of rhizosphere processes. They are also reported to control plant stress mechanisms through alteration of antioxidant systems and redox homeostasis, which reduces oxidative damage during unfavorable environmental conditions [12–14]. These attributes make fungi promising candidates for alleviating MP − induced stress in agricultural systems.

In addition to their role in stress mitigation, fungi have also been found to be able to interact directly with MPs in soils. Fungal hyphae may grow on the surface of plastic particles, which leads to the establishment of physical and biochemical interactions [15]. Some fungi also have extracellular enzymes that can alter complex polymers [16], resulting in some degradation or conversion of MPs. The processes can also add functional groups like carbonyl and carboxyls, which can change the physicochemical characteristics of MPs, including their hydrophobicity and interaction with soil particles [15, 17, 18]. Even though full mineralization of MPs has not been confirmed yet, these alterations could affect the environmental behavior and biological effects of the MPs in soil systems. The degree of contribution of these fungal − mediated processes to stress alleviation in plants is however not well understood.

Among the diverse fungal taxa, species of the genera Trichoderma and Metarhizium are of special interest because of their well − known contribution to agricultural and general environmental interactions. The Trichoderma (TD) species are well known due to their plant growth − promoting properties which include an increase in nutrient acquisition, root development, and systemic resistance against stress [19, 20]. Their possible role in transforming plastic − derived compounds has also been proposed by some studies, for instance, *Trichoderma harzianum* has shown potential in degrading low − density polyethylene (LDPE) MPs, with increased biomass and degradation of contaminants was observed [21]. However, the specific mechanisms by which TD interact with MPs in plant soil system, as well as the extent of degradation it can achieve, require further investigation. Similarly, Metarhizium (MET), an entomopathogenic species, have also been shown to interact with different environmental contaminants [22], implying a potential interaction with MPs. Despite these promising attributes, their specific roles (both TD and MET) in mitigating MP − induced stress and influencing MP behavior in plant–soil systems remain under-explored, particularly when applied individually or in combination. The knowledge gaps this study aims to address is the role of these fungal species (sole and in combination) in MPs induced stress conditions − to investigate the synergistic potential within the plant − soil system, especially their impact on plant health.

To address these knowledge gaps, it is essential to investigate plant–fungus–microplastic interactions within a well  defined experimental framework. In this context, maize (*Zea mays*) is chosen as the test/model crop because of its substantial relevance to agriculture and its widespread cultivation, and is already known sensitivity to environmental stressors, such as MPs [23]. Maize is particularly valuable for this study due to its extensive root system, as the roots are in direct contact with contaminants present in the soil, influencing plant’s health and growth and its ability to absorb nutrients, thus making it a reliable model for assessing the impacts of MPs in plant − soil systems.

This study focuses on PET-MPs as a persistent and prevalent soil contaminant, as its effect on plant − microbe − soil interactions remain inadequately explored. Moreover, studies are devoted to the problem of MP contamination, there is limited information about how beneficial fungi can regulate the plants’ reactions under the conditions of the MP stress and how these conditions can affect the soil health.

Therefore, it is hypothesized that fungal inoculation with TD and MET − sole or in combination – can mitigate the adverse effects of PET-MPs stress in maize, potentially by enhancing the plant physiological responses, including stress tolerance mechanisms, might results in improved growth. This study focuses on stress alleviation and plant–microbe interactions and does not evaluate PET−MPs degradation by the fungal strains. The primary objectives of this study are:

i. To evaluate the impact of PET-MPs on maize growth, oxidative stress responses and soil biological functions.
ii. To investigate the role of TD and MET inoculation in modulating antioxidant systems, redox balance, and metabolic responses in maize grown under PET-MPs-contaminated soil conditions.
iii. To assess the influence of fungal inoculation on rhizosphere microbial activity and soil enzyme responses under PET-MPs contamination

This study combines plant physiological responses with soil microbial dynamics and aims to provide a comprehensive understanding of how beneficial fungi can alleviate PET−MPs induced stress in agroecosystems. The findings are expected to contribute to the development of sustainable strategies for improving crop tolerance under stressed conditions and maintaining soil quality in environments increasingly affected by PET-MPs contamination.

## Materials and methods

### Materials and experimental procedure

#### Soil sampling and characterization

Soil samples were collected from the National Agriculture Research Center (NARC) at coordinates (33.6731913^o^N, 73.1212027^o^E), extracted from a depth interval of 0 − 15 cm. To ensure the representativeness, soil samples were collected from five random locations within the field – then pooled and homogenized to form a composite sample. Post − collection, the soil was expeditiously transported to the laboratory, securely stored in plastic bags. To reduce the risk of plastic contamination, the bags were rigorously cleaned prior to use, and all the handling procedures were carefully controlled to avoid any external contamination. The soil was first sieved through a 4 mm mesh to remove minor roots and extraneous debris. After sieving the soil was autoclaved at 121°C for 20 min to eliminate microbial contamination. Soil sterilization is commonly employed in controlled greenhouse experiments to minimize background microbial variability and to enable mechanistic assessment of introduced microbial inoculants under standardized conditions. This approach allows the functional contribution of inoculated fungi to soil enzyme activities and microbial community development to be evaluated without confounding effects from heterogeneous native microbiota and has been widely applied in plant–microbe interaction studies [24–26].

The soil demonstrates a texture characteristic of silt loam and originates from alluvial deposition. Pertaining to climatic attributes, the annual precipitation and mean temperature stand at 375 mm and 13.70 °C, respectively. The key soil properties are mentioned in Table S1. No external nutrient solutions were used throughout the experiment, as the nutrients present in the soil are adequate to support plant growth throughout the course of experiment. The collected soil had a pH of 7.2, which is considered neutral. Organic matter content was 0.69%, whereas other important soil properties included electrical conductivity (EC) were at 0.254 dS/m, phosphorus (P) at 4.45 mg/kg, potassium (K) at 16 mg/kg, boron (B) at 0.664 mg/kg, and nitrate nitrogen (NO₃–N) at 2.93 mg/kg.

### Microplastic (PET-MPs) preparation and characterization

PET-MPs employed in the present study were bought from Sigma − Aldrich, supplied as a white spherical powder with uniform appearance. Environmental MPs are often irregular in shape and heterogeneous in size, which introduces variability and complicates the interpretation of biological responses. The use of commercial uniform PET-MPs assures reproducibility and control over other particle characteristics such as size and shape. This enables a more focused study of the specific biological effects of their MPs, for instance, including physical properties (size, surface area) on interactions with plants and microbes [27]. In addition, uniform MPs are typically used in MP toxicity studies to isolate the effects of MP exposure without the confounding variability typically observed in environmental MPs [28–30]. Polyethylene terephthalate (PET) was selected as the model MP because it is one of the most persistent and frequently observex polymers in terrestrial environments, including agricultural soils impacted by irrigation water, packaging residues, and plastic fragmentation. Although polyethylene (PE) is commonly used in agricultural mulching, PET differs fundamentally from PE in density, rigidity, and surface chemistry, leading to distinct interactions with soil matrices, microorganisms, and plant roots [31, 32]. Therefore, MPs concentrations reported for PE cannot be assumed to be directly comparable to PET. In the present study, PET-MPs were applied on a mass − based basis to impose a defined MP load for mechanistic evaluation of plant − soil − microbe responses, rather than to infer polymer − specific equivalency or field − representative exposure levels.

**Fig. 1:**
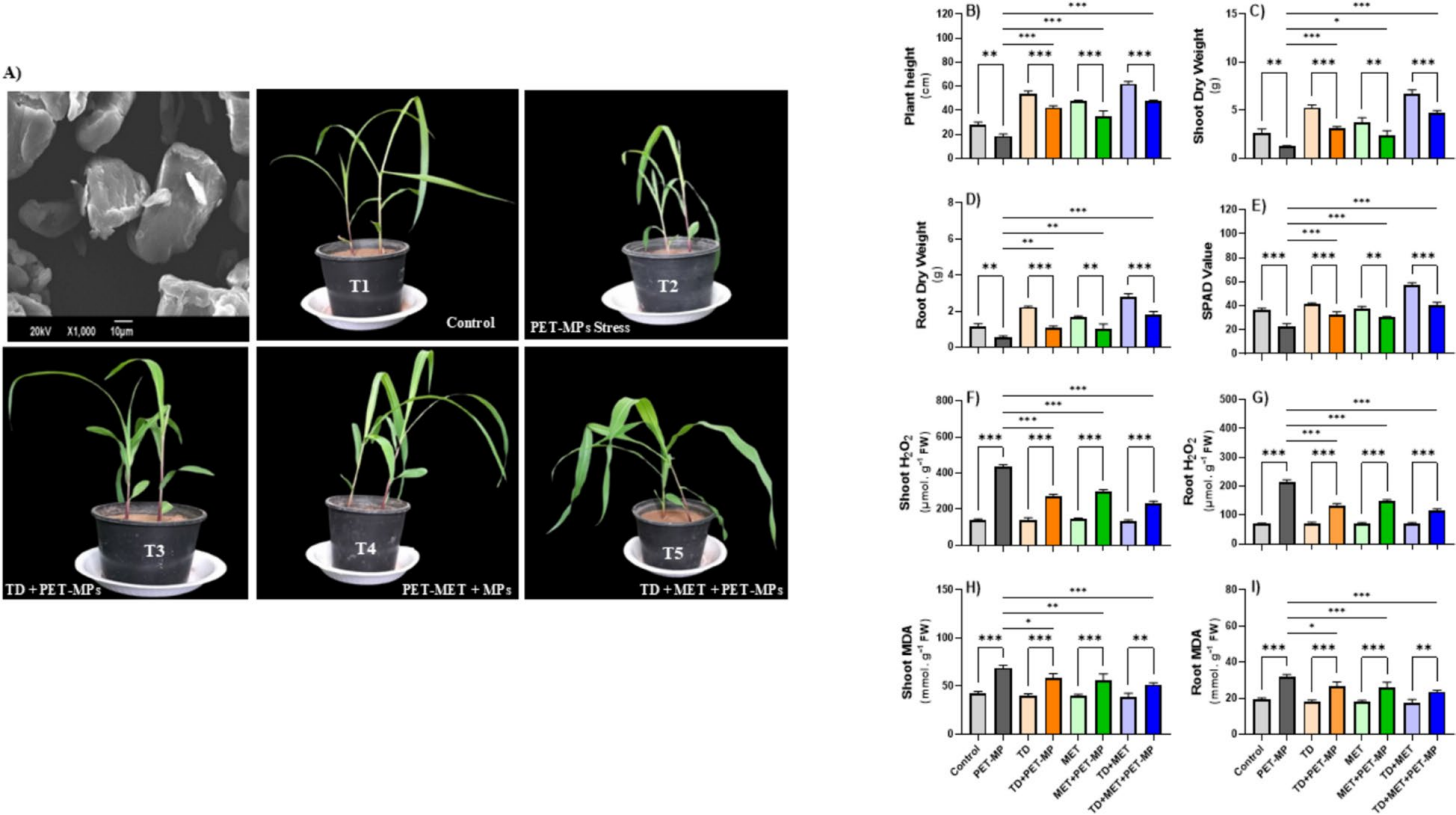
The figure depicts **A**) SEM image of PET-MPs and an experimental set − up shows maize plants consisting of control, PET-MPs and microbial inoculation treatment, **C** Plant height, **D** Shoot dry weight, **E** Root dry weight, **F** Soil Plant Analysis Development (SPAD) values, **G** Shoot Hydrogen Peroxide (H_2_O_2_), **H** Root H_2_O_2_, **I** Shoot Malondialdehyde (MDA) and **J**) Root MDA were analyzed in maize plants exposed to PET-MPs stressed conditions with and without fungal inoculation. The data were expressed as mean ± SD (*n* = 4). Statistical significance was indicated as *p < 0.05, **p < 0.01, and ***p < 0.001

A Scanning Electron Microscope (SEM, JEOL JSM − 6490 A, Analytical Scanning Electron Microscope) was used to determine the morphological aspects of the particles at an accelerating voltage of 20 kV and magnifications of 20,000 and 50,000. The SEM imaging of the PET particles demonstrated a rounded, spherical shape, and the particle size distribution ranged below 0.5 μm to 10 μm (Fig. 1A), which matched the size distribution of MPs environmentally relevant particles in polluted soils.

### Experimental design and greenhouse conditions

To investigate the impact of fungal isolates on PET-MPs in maize (*Zea mays* cv. CS220), we conducted a greenhouse experiment, over a period of 8 − weeks covering both early and mid − vegetative stages, that encompassed eight treatments, systematically arranged into distinct sets i) The first set, serving as the control group, remained untreated, ii) the second set of plants was subjected to stress induced by PET-MPs, iii) Sole Trichoderma application without PET-MPs stress iv) Trichoderma and PET-MPs stress, v) Sole Metarhizium application without PET-MPs stress, vi) Metarhizium and PET-MPs were introduced, vii) Combined Trichoderma and Metarhizium application without stress, and viii) a comprehensive treatment involving the combined application of Trichoderma, Metarhizium, and MPs were applied.

Each treatment consisted of 4 biological replicates (*n* = 4 pots per treatment), making a total of 32 experimental pots. Each pot (12 cm x 8 cm surface and 18 cm height) was loaded with 1 kg of autoclaved soil (dry weight). The pot dimensions and soil mass were selected to facilitate controlled assessment of early and mid − vegetative physiological responses rather than full − season crop development. Under such pot − based greenhouse conditions, root growth is physically constrained, which can limit nutrient acquisition, delay developmental progression, and suppress shoot elongation relative to field − grown maize. Therefore, although the experiment lasted eight weeks, plants remained within the vegetative growth phase, and plant height values reflect stress − and space − limited growth rather than normal tasseling − stage development.

Moreover, soil moisture was maintained at 65% of field capacity throughout the experiment. Soil moisture level was measured via gravimetric method (1) and adjusted as needed by adding the distilled water to the pots.

1 $$\text{Mositure Content}\;\%\;=\frac{\text{Wet weight}-\text{Dry weight}}{\text{Dry weight}}\times 100$$

where:

Wet weight = weight of the soil samples immediately after collection.

Dry weight = weight of the soil samples after drying in an oven at 105 °C for 24 h.

### Microplastic application and incubation

PET-MPs were added manually to soil by a thorough mixing to ensure a homogeneous mixing of the material. The PET-MPs were added in the concentration of 10% of the soil dry weight [33] − such elevated levels are commonly employed to assess threshold effects and mitigation efficacy, particularly where long − term field accumulation and heterogeneous PET-MPs distribution may result in localized high − load conditions. To get a uniform distribution, PET-MPs were first weighed and then sprinkled gradually onto the soil in a clean stainless − steel tray and soil and PET-MPs stirred up with a sterile metal spatula till a visually uniform mixture was obtained. After the addition, the soil was left to incubate under controlled conditions (to mimic natural conditions). The pots with the soil amended by MP were incubated for 1 week at 25 °C, with soil moisture maintained at ~ 65% of field capacity and pots covered to minimize evaporation, to allow homogenous incorporation of PET-MPs in the soil matrix before planting. This incubation period was able to equilibrate the PET-MPs within the soil so that they could interact effectively with the soil − plant system in the experiment phase.

### Seed sterilization and plant growth conditions

The seeds were sterilized to eliminate any potential contamination. Initially, they were treated with a solution consisting of 3 ml of sodium hypochlorite diluted with 97 ml of distilled water. Subsequently, the seeds are immersed in 100 ml of ethanol to further their sterilization, thereby eliminating any undesirable microorganisms present on the seed surface. Subsequently, the seeds undergo a thorough cleaning procedure, involving 4 to 5 washes with distilled water. Subsequently, the sterilized seeds were subjected to a germination period of 4 days. Two seeds of *Zea mays* were placed in each pot to minimize the border impact. On a weekly basis, the pots were rebalanced, and their locations were randomized. The seeds of maize were grown under the controlled conditions: day/night temperature of 26 °C/20 °C, relative humidity of 60%, day/night photoperiod of 14/10 h, and light intensity of 6000 lx [34].

### Fungal isolation and inoculum preparation

Strains of *Trichoderma longibrachiatum* (A8; acc. KY967258.1) and *Metarhizium anisopliae* (V245) were obtained from FIV − G ASAB, NUST, and the University of Swansea (UK), respectively The fungi were cultivated on potato dextrose agar (PDA) and the incubation period of the fungi was 6 days in the case of Trichoderma and 14 days in the case of Metarhizium. The synergistic response between the two strains were confirmed via plate experiment, Figure S2.

The harvesting of conidia was performed by placing sterile distilled water (3 mL) in each plate and scraping the surface. The suspension was filtered using Whatman No.3 filter paper and then processed through 0.22 − mm Millipore membranes to eliminate debris [35]. The concentration of spores was calibrated to 10^5^ − 10^8^ spore’s mL^−1^ and incubated at 4 °C. Viability was verified by subculture on PDA and counting of colonies after 48 h.

Each inoculation (sole or combined) were performed with a concentration of 1 × 10^7^ spores’ mL^−1^, whereas, similarly, for co-inoculation treatments, same conidial suspensions of TD and MET were prepared separately with equal volumes of both suspensions were mixed to obtain the co - inoculum, a total volume of 1 mL of this mixed suspension (containing 0.5 mL of TD and 0.5 mL of MET) were applied per plant at sowing, ensuring that each fungus was delivered at an equal proportion under co-inoculation conditions. The aliquot of conidial suspension 1 mL was applied to the soil at a depth of approximately 2 cm during sowing. The inoculation was done once at the beginning of the experiment. The enhanced parameters of plant growth such as root biomass, shoot height, and plant vigor were used to infer successful rhizosphere colonization (Fig. 1).

### Assessing hydrogen peroxide (H_2_O_2_) levels and lipid peroxidation

Determination of H_2_O_2_ levels in root and shoot samples was conducted following the method outlined by [36], with slight modifications. A fresh plant tissue (approximately 0.5 g) was homogenized by 0.1% (w/v) trichloroacetic acid (TCA) and then centrifuged at 12,000 × g and 4 °C for 15 min. The supernatant was combined with 10 mM potassium phosphate buffer (pH 7.0), and 1 M potassium iodide (KI). After the incubation (10 min) in the dark, absorbance was recorded at 390 nm. For each treatment, three biological replicates were analyzed to ensure statistically reliability.

Lipid peroxidation was assessed by the levels of malondialdehyde (MDA), as per the guidelines suggested by [37]. Fresh shoots and roots weighing 0.2 g were extracted (three biological replicates) in a 10 mL TBA − TCA solution (10% trichloroacetic acid with 0.25% 2 − thiobarbituric acid). The resulting mixtures were subjected to a 30 − minute incubation in boiling water, followed by immediate termination of the reactions in an ice bath. Supernatants were tested for absorbance at 532 nm after being centrifuged for 10 min at 10,000 xg. The amount of the MDA − TBA complex was calculated using an extinction coefficient of 155 L mmol^−1^ cm^−1^ after non − specific turbidity was subtracted by measuring at 600 nm, using the following formulae (3).

2 $$\text{Corrected absorbance}\;=\;\mathrm{A}_{532}\;-\;{A}_{600}$$

3 $$MDA concentration = \frac{(A_{532}-A_{600})}{155}$$

### Determination of ascorbic acid (AsA) and Dehydroascorbic acid (DHA) contents

Quantification of ascorbate levels followed the protocol established by [38], with slight modifications. Coarsely grinding the roots and fresh shoot samples (0.5 g) and extracting them with 2 mL of cold 6% (w/v) trichloroacetic acid (TCA). The resulting homogenates were centrifuged at 4 ℃ for 15 min at a speed of 10,000 g to remove any remaining debris. An equal volume of 30 mM phosphate buffered saline (PBS) with a pH of 7.4, 2.5% TCA, 8.4% H_2_PO_4_, 0.8% bipyridyl, and 0.3% FeCl_3_ (Ferric chloride) was added to a part of the supernatant. The combination was kept in a 40 °C oven for 30 min. The absorbance at 525 nm (AsA) was used to determine the amount of AsA. A portion of the supernatant was treated with 30 mM PBS containing 0.5 mM dithiothreitol (DTT). A mixture of N − ethylmaleimide, 2.5% TCA, 8.4% H_2_PO_4_, 0.8% bipyridyl, and 0.3% FeCl_3_ was put to a pot and heated for 15 min at 40 °C. DHA content was calculated as:

4 $$DHA\,=\,Total\,ascorbate\left(sum\,of\,AsA\,and\,DHA\right)\,-\,AsA$$

### Analyzing reduced glutathione (GSH) and oxidized glutathione (GSSG) levels

Glutathione levels were assessed using a fluorometric method developed by [39]. For the analysis, fresh shoot and root samples weighing around 1.0 g were finely refined and extracted in a mixture of 4.5 mL of 0.1 M phosphate − EDTA buffer (pH 5.0, containing 5 mmol L ^− 1^ EDTA) and 0.4 mL of 25% HPO_3_. Using the fluorescent probe O − phthalaldehyde, the presence of reduced glutathione (GSH) and oxidized glutathione (GSSG) in the resulting supernatant was determined after a 30 − minute centrifugation at 10,000 g at 4 °C. The detection process involved measuring GSH at pH 8.0 and GSSG at pH 12.0, leading to the formation of a substance that emitted light at a wavelength of 420 nm (350 nm excitation wavelength). N − ethylmaleimide was added at a final concentration of 30 mM to mitigate any interference caused by GSH in the GSSG determination [40].

### Quantification of enzymatic activities

The powdered roots and young shoots, weighing 0.3 g, were extracted using 3 mL of ice − cold 50 mM PBS (pH 7.8). The activity of antioxidant enzymes was quantified in the liquid portion of sample following centrifugation at 10,000 g for 15 min. The technique developed by Wu et al. was used to evaluate the activity of peroxidase (POD, EC 1.11.1.7), catalase (EC 1.11.1.6), and superoxide dismutase (SOD, EC 1.15.1.1). The procedures of glutathione reductase, glutathione peroxidase and ascorbate peroxidase activities were measured according to the procedure outlined by [41]. The enzymes mono − dehydroascorbate reductase (MDHAR, EC: 1.6.5.4) and dehydroascorbate reductase (DHAR, EC: 1.8.5.1) were analyzed using the techniques described by [42] and [41].

### Expression of genes via real − time polymerase chain reaction (qRT − PCR)

Fresh maize leaves (2 to 3 leaves per plant, each < 0.2 g) were picked and frozen in liquid nitrogen. TRIzol reagent was used to extract the total RNA. In short, frozen tissue was homogenized and mixed with TRIzol and then subjected to phase separation with chloroform, then RNA precipitated using ice − cold isopropanol. The ethanol − washed RNA pellet was air − dried and resuspended using nuclease − free water. The NanoDrop spectrophotometer was used to determine the quantity and purity of the RNA, where acceptable values were obtained on 260/280 and 260/230. A 20 uL reaction containing oligo(dT) primers, dNTPs, RNase inhibitor, and reverse transcriptase was used to make the first − strand cDNA with the RevertAid First Strand cDNA Synthesis Kit (Thermo Fisher Scientific) using 1 uL of total RNA. The reaction was incubated at 42 °C, 60 min. The quality of cDNA was checked by amplification of the housekeeping gene of actin (used as a reference gene confirming stability for reliable normalization in qRT − PCR analyses). The quantitative real − time PCR (qRT − PCR) was conducted with SYBR Green Master Mix (Fine Biotech) in 15 µL of the diluted cDNA (1:5) and gene − specific primers (Table S1). Amplification was performed on an Applied Biosystems StepOne system with an initial incubation of 94 °C 5 min and 40 cycles of incubation of denaturation (94 °C, 15 − 30 s), annealing (50 °C, 45 s) and extension (72 °C, 15 − 30 s). All the reactions were performed in triplicates. The 2 − ΔΔCT method was used to determine relative gene expression [43], with actin as the internal reference gene. Gel electrophoresis and melt curve confirmed primers specificity by displaying single and specific amplification products.

### Soil enzyme activity

Soil enzyme activities were measured in rhizosphere soil to capture microbial responses in the zone directly influenced by roots and root exudates under PET-MPs stress. To measure the activity of dehydrogenase (TPF mg kg^−1^ soil d^−1^) method as used as outlined by [44] was followed. Activity of urease (mg kg^−1^ soil d^−1^) was assessed by assessing the concentration of NH_4_^+^ released during the hydrolysis reaction, using a standard curve established with ammonium chloride [45]. The phosphatase activity (mg kg⁻^1^ h⁻^1^) was assessed by using the substrate p − nitrophenyl phosphate disodium (0.115 M). Prior to analysis, soil samples were sieved to a particle less than 2 mm, sodium acetate buffer (0.5 M) solution was then used with 2 mL of the sample, and the 6 pH was maintained by addition of acetic acid, ensuring its maintenance throughout the experiment [46] following that, the substrate (0.5 mL) and the sieved soil (0.5 g) were combined, resulting mixture was incubated at 37 °C for 90 min. To stop the reaction, the mixture was chilled to 0 °C and held there for 10 min. The soil was treated with 0.5 mL of 0.5 M CaCl_2_ and 2 mL of 0.5 M NaOH. The resulting mixture was then centrifuged at a speed of 4000 revolutions per minute for a duration of 5 min. The concentration of PNP was determined using spectrophotometry at a 398 nm [47]. The spectrophotometric method for measuring soil sucrase activity was 3, 5 − dinitrosalicylic acid. The activity of soil − sucrase was quantified as enzyme activity units (ml g^−1^ soil h^−1^). To determine the catalase activity (ml g^−1^ soil h^−1^) protocol of [48] was followed, the remaining H_2_O_2_ in the filtrate was then titrated with 0.2 mol L^−1^ KMnO_4_ solutions and was expressed as 0.2 mol L^−1^ KMnO_4_ ml g^−1^ h^−1^. 3, 5 − dinitrosalicylic acid colorimetric method by (Guan 1986) was utilized to determine the amylase activity. The activities of sucrase and amylase were measured in mg kg^−1^ h^−1^of glucose and maltose, respectively. For each enzyme assay, substrate blanks (containing substrate but no soil) and soil blanks (soil without substrate) were included to correct for non − enzymatic reactions, ensuring that measured activity reflects true microbial enzymatic activity.

### Phospholipid fatty acid analysis of microbial communities

Phospholipid fatty acid (PLFA) analysis was carried out following a modified Bligh and Dyer extraction method [49]. Soil samples (6 g fresh weight) were extracted with a chloroform: methanol: citrate buffer (1:2:0.8, v/v/v). The internal standard was 19:0 nonadecanoic acid. Lipids were separated by phase partitioning after centrifugation and fractionated by silica solid phase extraction. The phospholipids were saponified in a sodium hydroxide solution and then methylated using a methanolic NaOH solution and 12% boron trifluoride to form fatty acid methyl esters (FAMEs). The organic phase was extracted with hexane and 5α − cholestane was used as an additional internal standard. These were solvent − washed, dissolved in isooctane and injected into a GC 2010 gas chromatograph (Shimadzu, Japan) with a flame ionization detector and a capillary SPB − 5 column (30 m × 0.25 mm × 0.25 µm). PLFA contents were calculated on a dry weight basis using external standards. Microbial biomass and community structure were determined by using known PLFA biomarkers for major groups, such as Gram − positive bacteria, Gram − negative bacteria, actinomycetes and fungi. Since PLFA biomarkers are not strictly unique to specific groups, the data were used to infer community structure rather than specific taxonomic composition [49–52].

### Microbial activity

A soil sample weighing 15 g underwent a 24 − hour fumigation process at a temperature of 25 °C using chloroform, specifically ethanol − free chloroform. Following this, samples were subjected to extraction using a solution of 0.5 M K_2_SO_4_. The identical procedures were carried out on a separate soil sample that had not been subjected to fumigation. After the process of titration Microbial Biomass Carbon (MBC) was calculated as mentioned by [53]. MBC was calculated using the correction factor (kEC) of 2.64 to account for the portion of C released during fumigation. The total nitrogen content in the potassium extract was determined using the Kjeldahl digestion method [54]. Briefly, the soil extract was digested with the digestion mixture and heated up. The resulting mixture was cooled and distilled and titrated with 50 mM sulfuric acid. Blank controls have been run to consider the background nitrogen. Standard ammonium chloride solutions were used to calibrate the titration method for accuracy and reliability of the results. The ammonium (NH_4_^+^) and nitrate (NO_3_^−^) concentration in the rhizosphere was determined with a continuous flow analyser (Skalar SAN + + System, Skalar Analytical B.V., Breda, Netherlands). Microbial Biomass Nitrogen (MBN) was calculated using the correction factor (kEN) of 0.45 to adjust for nitrogen released during fumigation. MBN was calculated by the following formula,

5 $$\mathrm{MBC}\,=\,\left(\text{Carbon fumigated}\;-\;\text{Carbon non-fumigated}\right)\times1.4$$

The Dissolved Organic Carbon (DOC) present in the rhizosphere soil was extracted using a 0.5 M K_2_SO_4_ solution in a ratio of 3 g of soil to 15 ml of the extracting solution. The mixture was then subjected to shaking for a duration of 1 h. Subsequently, the extract was filtered using a quantitative filter [55] and the filtered solution was analyzed using a total organic carbon analyzer (Multi − N/C 2100 S; Analytik Jena, Jena, Germany) whereas formula for MBC is.

6 $$\mathrm{MBC}\;=\;(\mathrm{Carbon}\;\mathrm{fumigated}\;-\;\mathrm{Carbon}\;\mathrm{non}-\mathrm{fumigated})\;\mathrm x\;2.64$$

### Root exudate profiling and quantification of organic compounds

Root exudates were collected from maize plants at the early to mid − vegetative growth stage (V4–V6), when exudation is metabolically active [56, 57]. Roots were washed with sterile water and then plants were incubated for 24 h in sterile water under controlled growth conditions. Three plants of each treatment were pooled together, filtered using 0.22 µm syringe filters, and lyophilized. These samples were reconstituted in methanol (1 mg/mL), vortexed (1 min) and solvent is removed under vacuum at 40 °C for 90 min then 70ul of N − methyl − N − (trimethylsilyl) trifluoroacetamide (MSTFA) is added to the sample and incubated at 40 °C, 30 min to silylate the residues.

GC − MS analysis, the prepared samples were taken to the US − Pakistan Center for Advanced Studies in Energy (USPCAS − E), NUST, where derivatization and analysis processes were done. GC − MS analysis was performed by using a Shimadzu GC − 2010, equipped with flame ionization detector (FID) and a SH − Rxi − 5Sil MS fused silica capillary column (30 m × 0.25 mm × 0.25um film thickness). The oven temperature was programmed to an initial temperature of 50 °C, which was maintained for 1 min, followed by elevation at 10 °C/min to 320 °C and maintenance for 10 min. Helium was used as the carrier gas with a constant flow rate of 1 mL/min. The mass spectra were recorded in full scan mode (m/z = 50 − 550). The metabolites were detected using the NIST library and were measured using standard calibration curves (mg/plant/day) including sugars, sugar alcohols, fatty acids, and organic acids.

In addition to GCMS profiling, the amounts of total organic carbon (TOC) and total carbohydrates were measured in root exudates to study how MPs caused changes in carbon exudation. TOC was determined using a catalytic combustion method coupled with NDIR detection, using procedures described by [58]. Results for total carbohydrate were obtained by measuring absorbance at 490 nm, using the color − test phenol–sulfuric acid method and a glucose − based standard curve [59].

### Statistical analysis and multivariate analysis

The data gathered underwent statistical analysis. To perform statistical testing, it was used to run the Statistix 8 software, and to perform data visualisation with the help of the GraphPad Prism. The Shapiro Wilk test was performed to evaluate data normality, and the test of equal variance was performed by means of the Levene test. In cases where variables displayed non − normal distributions or unequal variances, logarithmic transformations were applied to address these issues.

The statistics are provided as means ± standard errors (SE), which are obtained based on three independent sets of data, and these values are available in Table S2. The effect size for each comparison between treatment groups was calculated using Cohen's d, which quantifies the standardized difference between the means of two groups. The calculation of Cohen d was based on the formula (7):

7 $$d= \frac{{M}_{1}- {M}_{2}}{{SD}_{pooled}}$$

where, M1 and M2 are the means of two being compared (e.g., Control vs MP), and SD _pooled_ is the pooled standard deviation calculated as:

8 $${SD}_{pooled }=\sqrt{\frac{({n}_{1 }- 1)\times {SD}_{1}^{2}+ ({n}_{1 }- 2)\times {SD}_{2}^{2}}{{n}_{1}+ {n}_{2 }-2}}$$

Here, n_1_ and n_2_ are the sample size of two groups and SD_1_ and SD_2_ are the standard deviations of two groups. The calculated size effects are present in Table S3.

Furthermore, to determine the synergy between the microbial inoculations (TD and MET) the Bliss Independence Model was employed in R studio 4.5.1. This model calculates the synergy between the microbial inoculation via the following formula (8), and is the values are presented in Table S3.

9 $${E}_{Bliss\;additive}= {T}_{1}+{T}_{2}-({T}_{1}\times {T}_{2})$$

In this case, E is the expect, T_1_ represents TD and T_2_ represent MET. The observed effect under each treatment was then compared to the expected effect. In R, the computation of expected and observed effects was performed through use of dplyr package, whereas expected effects were computed based on the “mutate ()” function and the findings were thus categorized.

Furthermore, the Tukey's (HSD) test was used to compare the average results across different treatments. Statistical significance was observed when the difference was equal to or less than 0.05. Moreover, structural equation modeling was performed using Phyton, while principal component analysis (PCA) was conducted using the ClustVis software (https://biit.cs.ut.ee/clustvis). The Chi − square (X^2^) test and Bentler's comparative fit index (CFI), which offers extra guidelines for model fit, were employed to test the model's correctness. A good match for the model is defined as a value greater than 0.90 [60, 61]. Another model fit statistic is root − mean − square error of approximation (RMSEA) was employed, which should be less than 0.10 [60, 61].

## Results

### PET-MPs impair maize growth and induce oxidative stress, effects ameliorated by microbial inoculation

PET-MPs stress exerted a considerable effect on maize growth, significantly reduced the plant height (−33%), shoot dry weight (−22%), root dry weight (−33%), and SPAD (−38%), relative to the non − stressed control (Fig. 1B–E). This was accompanied by elevated oxidative stress markers, with increased H₂O₂ (+ 209% in shoot; + 213% in root) and MDA (+ 63% in shoot; + 66% in root), compared to control (no PET-MPs; Fig. 1F–J).

Fungal inoculation (TD + MET > TD > MET) alleviated these negative effects, compared to stressed control (with PET-MPs). TD improved maize growth and reduced the oxidative stress markers, while MET showed moderate recovery in both physiological traits and oxidative status, relative to control (with PET-MPs; Fig. 1B–E). Co-inoculation (TD + MET) showed the strongest effect, significantly restored plant height (+ 193%), shoot dry weight (+ 93%), root dry weight (+ 107%), and SPAD (+ 78%), while notably reduced oxidative stress markers (H₂O₂ − 46% in both shoot and root; MDA − 25% in shoot; − 26% in root), relative to stressed control (with PET-MPs; Fig. 1F–J).

### PET-MPs induced oxidative stress alters antioxidant enzyme activity and gene expression

**Fig. 2:**
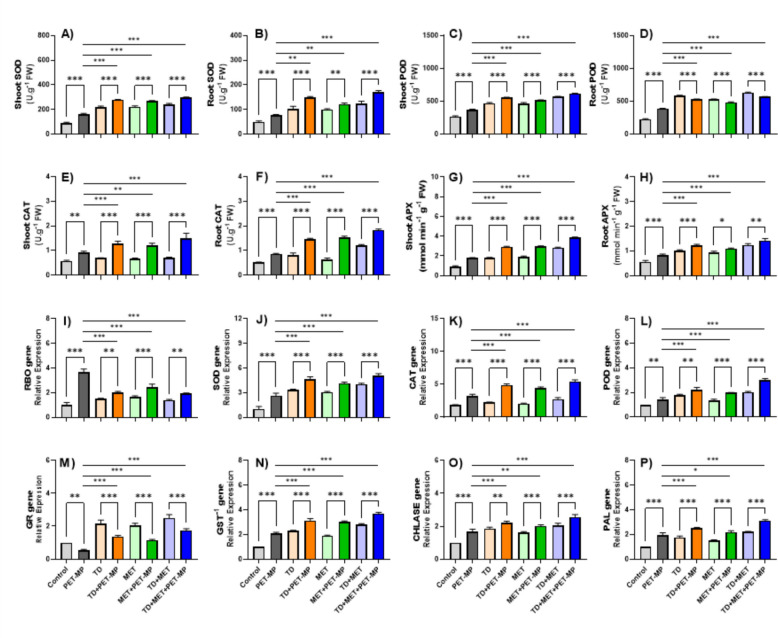
This figure depicts the analysis of antioxidant enzyme levels including Superoxide Dismutase (SOD) in shoot (**A**) and root (**B**), Peroxidase (POD) in shoot (**C**) and root (**D**), Catalase (CAT) in shoot (**E**) and root (**F**), and Ascorbate Peroxidase (APX) in shoot (**G**) and root (**H**). The figure also shows the levels of transcriptional expressions for Respiratory Burst Oxidase (RBO) gene (**I**), SOD gene (**J**), CAT gene (**K**), POD gene (**L**), Glutathione Reductase (GR) gene (**M**), Glutathione S − Transferase − 1 (GST − 1) gene (**N**), Chalcone Synthase (CHALASE) gene (**O**), and Phenylalanine Ammonia Lyase (PAL) gene (**P**) in maize plants under PET-MPs stress conditions with and without fungal inoculation. The data is presented as mean values ± standard deviation (SD), and statistical significance is marked as *p < 0.05, **p < 0.01, and ***p < 0.001, while "ns" indicates non − significant differences

The presence of PET-MPs had a significant impact on the levels of antioxidant enzymes in both the shoot and root of maize plants, relative to control (no PET-MPs; Fig. 2A − H). PET-MPs stress significantly increased the levels of antioxidant enzymes in both the shoot and the root where SOD, POD, CAT, and APX, substantially increased up to + 98% in shoot and + 73% in root, compared to control (no PET-MPs; *p* < *0.05*; Fig. 2A − H). Microbial inoculation, particularly the combined inoculation (TD + MET) significantly reduced oxidative stress with the significant increase in the enzyme antioxidant system such as, SOD (+ 50% in shoot, + 168% in root), POD (+ 53% in shoot, + 46% in root), CAT (+ 65% in shoot, + 113% in root) and APX (+ 118% in shoot, + 59% in root), respectively, compared to control (with PET-MPs; Fig. 2A − H).

Furthermore, the enzymatic modulations in maize plants corresponded to PET-MPs induced-stress, variations in the expression of antioxidants and stress-responsive genes showed a coordinated molecular response to oxidative stress, relative to control (no PET-MPs; Fig. 2I − P). PET-MPs also exhibited the upregulation of antioxidant and stress-responsive genes, such as Respiratory Burst Oxidase (RBO) (+ 267%), SOD (+ 163%), CAT (+ 223%), Glutathione S − transferas (GST^−1^) (+ 106%), chlorophyllase (CHLASE) (+ 173%) and Phenylalanine Ammonia − Lyase (PAL) (+ 93%) and suppression of Glutathione Reductase (GR) (− 43%), respectively, relative to control (no PET-MPs; Fig. 2I − P). TD and MET inoculation increased expression of these genes, with the combined TD + MET treatment showed the substantial increase in SOD (+ 92%), CAT (+ 68%), POD (+ 109%), GR (+ 341%) and GST ^−1^ (+ 66%) and a downregulation of RBO (− 47%) and CHLASE (− 10%), relative to control (with PET-MPs; *p* < *0.05*; Fig. 2I − P).

### Synergistic enhancement of ASA/DHA ratio by fungal inoculation in PET-MPs − stressed maize

**Fig. 3:**
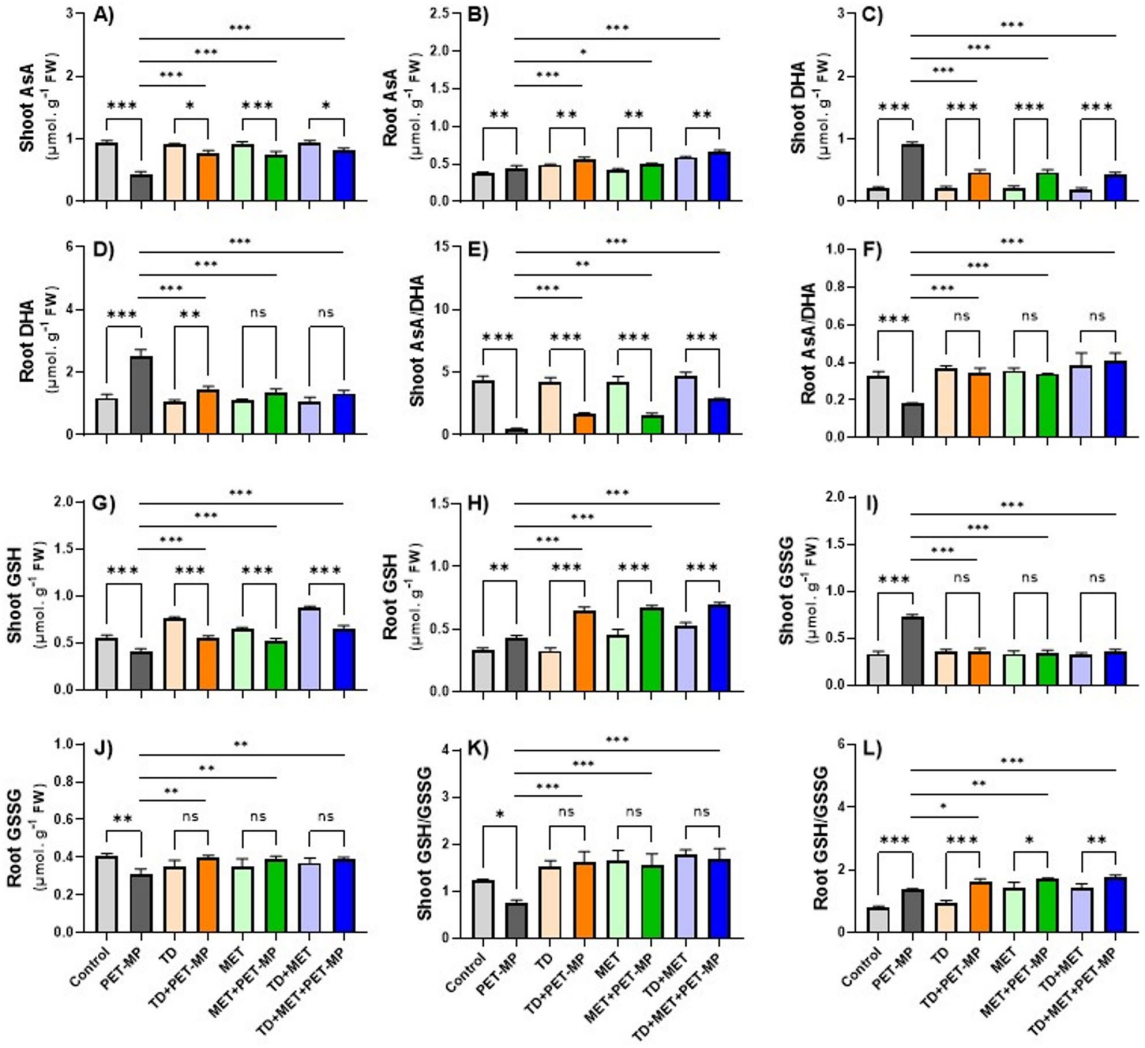
This figure shows the analysis of various antioxidant parameters, including the levels of ascorbic acid (ASA) in the shoot (**A**),and in root (**B**), dehydroascorbic acid (DHA) in the shoot (**C**), and in root (**D**), shoot ASA/DHA ratio (**E**), root ASA/DHA ratio (**F**), Glutathione (GSH) in the shoot (**G**), GSH in the root (**H**), Reduced Glutathione (GSSG) in the shoot (**I**), GSSG in the root (**J**), and the GSH/GSSG ratio in the shoot (**K**) and root (**L**). The data is presented as mean values ± standard deviation (SD), and statistical significance is indicated by *p < 0.05, **p < 0.01, and ***p < 0.001, while "ns" denotes non − significant differences

PET-MPs exposure markedly disrupted the ascorbate–glutathione redox system in maize, relative to control (no PET-MPs; Fig. 3A–L). A significant decrease in AsA was observed in shoots (− 54%), while roots showed a slight increase (+ 16%), accompanied by a significant accumulation of DHA (+ 319% in shoots; + 112% in roots), compared to non − stressed control (no PET-MPs; Figure. A − D). Consequently, the AsA/DHA ratio were drastically decreased (− 89% in shoots; − 45% in roots), relative to control (no PET-MPs; Fig. 3E − F).

Fungal inoculation mitigated these negative effects, relative to control (with PET-MPs; Fig. 3A − L). TD and MET both enhanced AsA levels (TD: + 81% shoot, + 24% root; MET: + 71% shoot, + 2% root) and reduced DHA (TD: − 49% shoot, − 42% root; MET: − 48% shoot, − 46% root), with co-inoculation showing the strongest restoration (AsA: + 91% shoot, + 21% root; DHA: − 53% shoot, − 47% root), relative to stressed control (with PET-MPs; Fig. 3A–D). This recovery was also indicated by the AsA/DHA ratio, which rose considerably under TD (+ 255% shoot and + 232% root), MET (+ 91% in both tissues), and most significantly under co-inoculation (+ 305% shoot; + 128% root), compared to control (with PET-MPs; Fig. 3E–F).

Likewise, PET-MPs altered glutathione dynamics, reduced GSH in shoots (− 25%) but increased in roots (+ 29%), while also notably reduced GSSG levels (− 54% shoot; − 24% root), resulting in higher GSH/GSSG ratio (+ 64% shoot; + 69% root), relative to control (with PET-MPs; Fig. 3G–L). Fungal treatments significantly enhanced both GSH (TD: + 35% in shoot, + 52% in root; MET: + 25% in shoot, + 57% in root) and GSSG levels, with most significant increase due to co-inoculation (GSH: + 42% in shoot, + 63% in root; GSSG: + 5% in shoot, + 26% in root), relative to control (with PET-MPs). The GSH/GSSG ratio was further enhanced in TD (+ 30% in shoot; + 19% in root), MET (+ 25% in shoot; + 19% in root) and TD + MET (+ 36% in shoot; + 30% in root), compared to control (with PET-MPs; Fig. 3K–L).).

### Fungal inoculation modulates the ascorbate − glutathione cycle under PET-MPs stress in maize

**Fig. 4:**
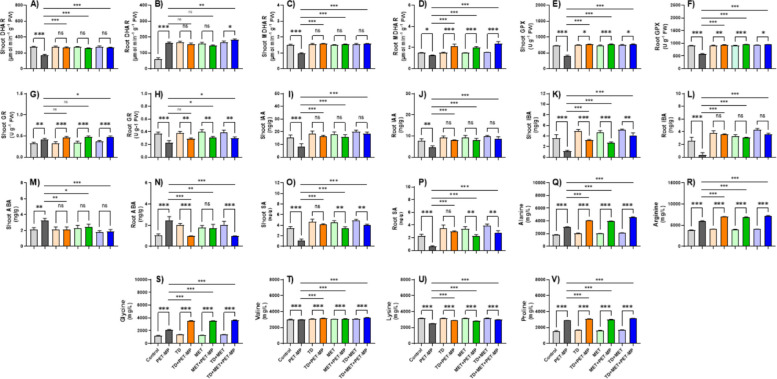
This figure represents the analysis of antioxidant enzyme levels, including Dehydroascorbate Reductase (DHAR) in shoot (**A**) and root (**B**), Monodehydroascorbate Reductase (MDHAR) in shoot (**C**) and root (**D**), Glutathione Peroxidase (GPX) in shoot (**E**) and root (**F**), Glutathione Reductase (GR) in shoot (**G**) and root (**H**), Indole-3-Acetic Acid (IAA) in shoot (I) and root (J), Indole-3-Butyric Acid (IBA) in shoot (K) and root (**L**), Abscisic Acid (ABA) in shoot (**M**) and root (**N**), and Salicylic Acid (SA) in shoot (**O**) and root (**P**). Additionally, the profiles of key amino acids — alanine (**Q**), arginine (**R**), glycine (**S**), valine (**T**), lysine (**U**), and proline (**V**) of maize plants. The data is presented as mean values ± standard deviation (SD), and statistical significance is indicated as *p < 0.05, **p < 0.01, and ***p < 0.001, while "ns" signifies non − significant differences

PET-MPs exposure significantly disrupted antioxidant systems, phytohormones, and metabolic profiles in maize, relative to control (no PET-MPs; Fig. 4A–V). In shoots, PET-MPs notably reduced DHAR (− 37%), MDHAR (− 35%), and GPX (− 43%), while significantly increased GR (+ 30%), compared to non − stressed control (no PET-MPs; Fig. 4A,C,E,G). In roots, DHAR (− 1%), MDHAR (− 38%), and GR (− 56%) were considerably reduced, whereas an opposite trend was observed for GPX (+ 19%), compared to control (no PET-MPs; Fig. 4B,D,F,H). Concurrently, PET-MPs markedly altered hormonal balance, considerably reduced IAA (− 44% in shoot; − 41% in root) and IBA (− 67% in shoot; − 77% in root), while significantly increased ABA (+ 55% in shoot; + 108% in root) and notably decreased SA (− 51% shoot; − 70% root), compared to non − strssed control (no PET-MPs; Fig. 4I–P). Metabolically, PET-MPs significantly induced accumulation of amino acids, including alanine (+ 65%), arginine (+ 56%), glycine (+ 72%), and proline (+ 85%), relative to control (no PET-MPs; Fig. 4Q–V).

Fungal inoculation mitigated these negative effects (TD + MET + PET-MPs > TD + PET-MPs > MET + PET-MPs), under PET-MPs stress. TD significantly increased shoot DHAR (+ 56%), MDHAR (+ 60%), GPX (+ 85%), and GR (+ 11%), while in roots it notably enhanced MDHAR (+ 46%), GPX (+ 16%), and GR (+ 26%) but slightly reduced DHAR (− 4%), relative to stressed control (with PET-MPs; Fig. 4A–H). Similarly, MET considerably increased shoot DHAR (+ 50%), MDHAR (+ 56%), GPX (+ 88%), and GR (+ 13%), and in roots notably enhanced MDHAR (+ 36%) and GPX (+ 32%) but reduced DHAR (− 10%), compared to control (with PET-MPs; Fig. 4A–H). Co-inoculation (TD + MET) showed a synergistic response, increased DHAR (+ 55% in shoot; + 13% in root), MDHAR (+ 61%; + 60%), GPX (+ 86%; + 48%), and GR (+ 15%; + 30%), relative to control (with PET-MPs; Fig. 4A–H).

In parallel, fungal application also restored hormonal balance, relative to control (with PET-MPs). TD significantly increased IAA (+ 94% in shoot; + 95% root) and IBA (+ 231% shoot; + 316% root), reduced ABA (− 36% in shoot; − 62% in root), and enhanced SA (+ 191% in shoot; + 335% in root), relative to stressed control (with PET-MPs; Fig. 4I–P). Furthermore, MET showed similar but slightly lower effects on IAA (+ 85%; + 88%) and IBA (+ 210%; + 270%), with partial ABA reduction and SA recovery, relative to control (with PET-MPs). Whereas, the combined TD + MET treatment showed the strongest response, restoring IAA (+ 115% in shoot; + 116% in root) and IBA (+ 290% in shoot; + 350% in root), while notably reduced ABA (− 30% shoot; − 60% root), whereas significantly increased SA (+ 200% shoot; + 400%) levels, relative to control (with PET-MPs − Fig. 4I–P). Additionally, co-inoculation further enhanced amino acid accumulation, increased alanine (+ 50%), arginine (+ 20%), glycine (+ 70%), and proline (+ 10%) levels, compared to control (with PET-MPs; Fig. 4Q–V).

### Root metabolic profiles reveal PET-MPs toxicity and microbial amelioration

**Fig. 5:**
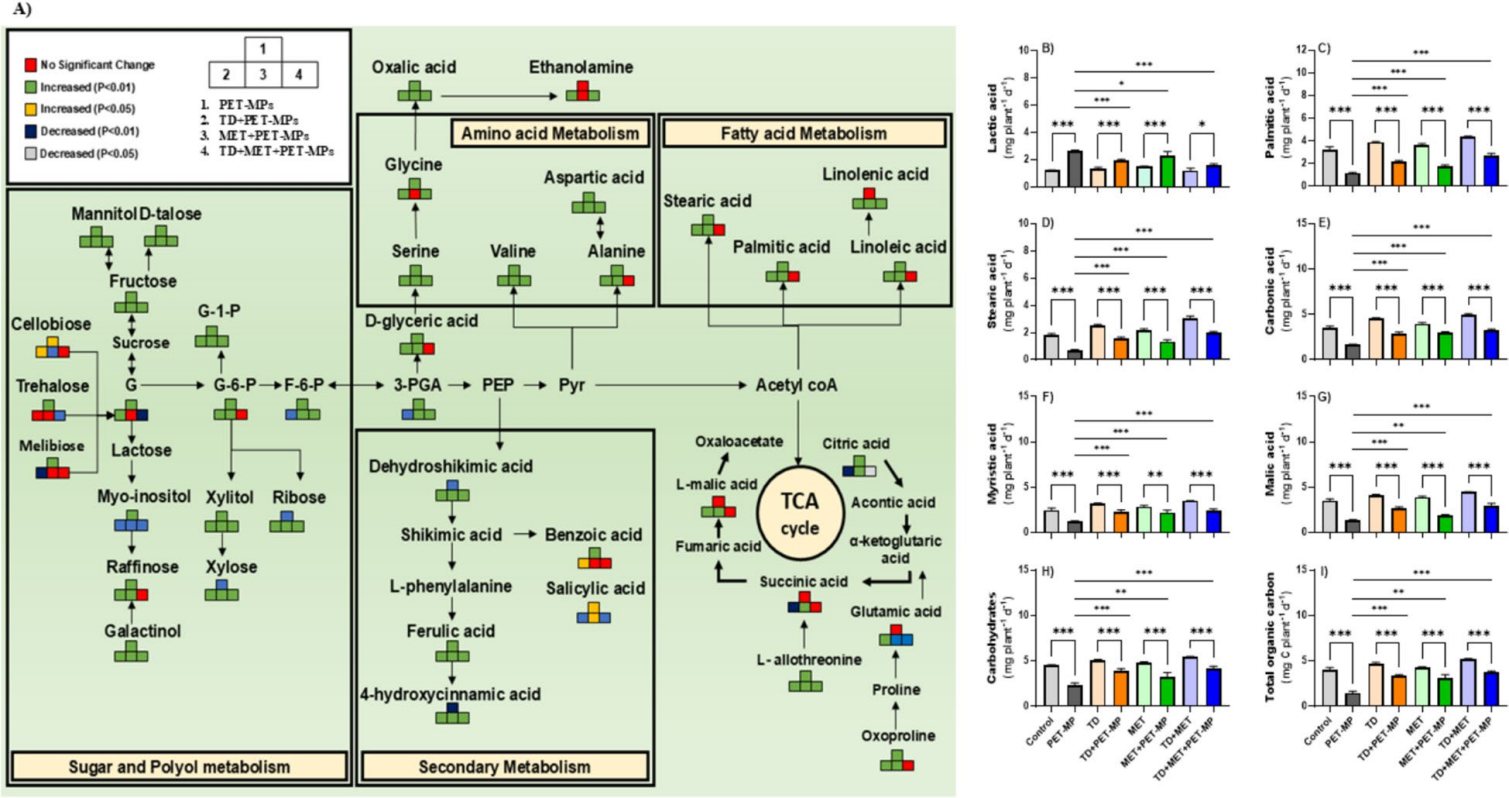
**A** Overview of altered metabolic pathways in maize root exudates based on untargeted GC − MS analysis under different treatments: MPs alone, TD + PET-MPs, MET + PET-MPs, and TD + MET + PET-MPs). Pathways include sugar and polyol metabolism, amino acid metabolism, fatty acid metabolism, the tricarboxylic acid (TCA) cycle, and secondary metabolism. Colored boxes indicate a statistically significant increase or decrease in metabolite levels relative to control (no PET-MPs), with red, yellow, blue, and green corresponding to PET-MPs, TD + PET-MPs, MET + PET-MPs, and TD + MET + PET-MPs, respectively. **B**–**I** Quantitative analysis of selected target root exudate metabolites: **B** lactic acid, **C** palmitic acid, **D** stearic acid, **E** carbonic acid, **F** myristic acid, **G** malic acid, **H** total carbohydrates, and (**I**) total organic carbon (TOC). Data are presented as mean ± SD (*n* = 3), and statistical significance was determined using one − way ANOVA followed by Tukey’s post hoc test (***p < 0.001, **p < 0.01, *p < 0.05, ns = not significant). Fungal inoculation, particularly T + M + PET-MPs, effectively restored key metabolite levels disrupted by PET-MPs stress

PET-MPs stress significantly disrupted maize root exudate metabolism patterns across primary and secondary pathways, compared to non − stressed control (no PET-MPs). Exposure of PET-MPs notably increased polyols and sugars including mannitol − talose, fructose, cellobiose, and melibiose, while reduced key metabolites such as sucrose, myo − inositol, and raffinose, relative to control (no PET-MPs; Fig. 5A). This change was accompanied by a decline in glycolysis and TCA cycle intermediates, including glucose − 1 − phosphate, pyruvate, and citric acid, relative to control (no PET-MPs). Furthermore, secondary metabolism was also altered, with notable increase in oxalic acid, aspartic acid, alanine, palmitic acid, and oxoproline, and a considerable reduction in ethanolamine and shikimic acid were also observed, compared to control (no PET-MPs). These metabolic disruptions were further reflected in root exudate composition, substantially reduced lactic acid by − 46% and significantly increased palmitic acid (+ 132%), stearic acid (+ 190%), myristic acid (+ 94%), carbonic acid (+ 96%), malic acid (+ 111%), carbohydrates (+ 77%), and total organic carbon (+ 155%), respectively, compared to control (with PET-MPs; Fig. 5B − I).

Fungal inoculation mitigated the negative effect of PET-MPs on root exudate metabolism in a coordinated manner, relative to control (with PET-MPs). Sole inoculation (TD > MET) restored metabolic balance by increasing sucrose and myo − inositol, reducing polyols (mannitol − talose and fructose), and enhancing glycolysis and TCA intermediates, relative to control (with PET-MPs; Fig. 5 A). They also downregulated stress − associated metabolites such as oxalic acid, aspartic acid, and alanine, while notably increased ethanolamine, shikimic acid, and linolenic acid, compared to control (with PET-MPs). However, the strongest response was observed under TD + MET co-inoculation, which resulted in the most effective recovery of sugar metabolism, polyol balance, glycolysis and TCA cycle intermediates, and secondary metabolites compared to control (with PET-MPs). This combined treatment also markedly improved root exudate composition by reducing lactic acid (− 46%) and restoring palmitic, stearic, carbonic, myristic, and malic acids, along with total carbohydrates and total organic carbon, compared to control (with PET-MPs; Fig. 5B–I).

### Effects of PET-MPs and fungal inoculation on soil chemistry, enzyme activity, and microbial community

**Fig. 6:**
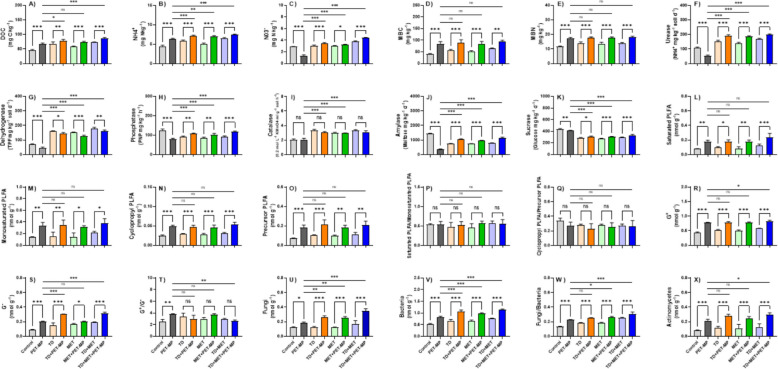
This figure shows the analysis of soil and microbial parameters, including Dissolved Organic Carbon (DOC) in panel (**A**), Ammonium (NH_4_^+^) in panel (**B**), Nitrate (NO_3_^−^) in panel (**C**), Microbial Biomass Carbon (MBC) in panel (**D**), Microbial Biomass Nitrogen (MBN) in panel (**E**), Urease activity in panel (**F**), Dehydrogenase activity in panel (**G**), Phosphatase activity in panel (**H**), Catalase activity in panel (**I**), and Sucrase activity in panel (**J**). Additionally, microbial community composition is represented by the levels of Saturated Phospholipid Fatty Acids (PLFA) (**L**), Monosaturated PLFA (**M**), Cyclopropyl PLFA (**N**), Precursor PLFA (**O**), and Cyclopropyl precursor PLFA (**P**). The figure also includes the analysis of microbial groups, such as Gram − positive (G +) (**Q**), Gram − negative (**G** −) (**R**), Bacteria (U), Fungi (**V**), Actinomycetes (**W**), and the Fungi/Bacteria ratio (**X**). The data is presented as mean values ± standard deviation (SD), and statistical significance is indicated by *p < 0.05, **p < 0.01, and ***p < 0.001, while "ns" denotes non − significant differences

Exposure to PET-MPs significantly altered soil chemistry, microbial community, and root exudate variables, relative to non − stressed control (no PET-MPs; Fig. 6A − H). PET-MPs significantly increased DOC (+ 49%), NH_4_^+^ (+ 19%), while notably decreased NO_3_^─^ (− 55%), relative to control (no PET-MPs; Fig. 6A − C). PET-MPs also considerably elevated MBC (+ 110%) and MBN (+ 49%), relative to control (no PET-MPs; Fig. 6D − E). Among soil enzymes, urease, phosphatase, amylase and sucrase activities were significantly reduced, while dehydrogenase and catalase showed moderate increase, relative to non − stressed control (no PET-MPs; Fig. 6F − K). Moreover, a shift in microbial community composition is also observed, with saturated PLFA (+ 126%), monounsaturated PLFA (+ 131%), cyclopropyl PLFA (+ 96%), and precursor PLFA were significantly increased, whereas the ratios of saturated/monounsaturated and cyclopropyl/precursor PLFA remained unchanged, relative to control (no PET-MPs; Fig. 6L − Q). Furthermore, PET-MPs exposure also increased gram − positive (+ 76%), gram − negative bacteria (+ 124%) and fungal abundance (+ 56%), and actinomycetes (+ 152%), compared to control (no PET-MPs; Fig. 6S − X).

Fungal inoculation significantly mitigated the negative effects of PET-MPs, compared to stressed control (with PET-MPs; Fig. 6A − X). Sole inoculation of TD and MET partially restored DOC, NH_4_^+^, NO_3_^─^, MBC, and MBN, whereas combined TD + MET inoculation produced the significant impact, increased DOC (+ 13%), NH_4_^+^, (+ 19%) MBC (+ 10%), MBN (+ 5%), and also restored NO_3_^─^ (+ 167%), relative to stressed control (with PET-MPs; Fig. 6A − E). Furthermore, TD + MET significantly enhanced urease (+ 274%), dehydrogenase (+ 260%), phosphatase (+ 50%), catalase (+ 43%), and amylase (+ 208%), whereas partially reduced sucrase activity, relative to stressed control (with PET-MPS; Fig. 6F − K). Moreover, fungal inoculation notably improved microbial community composition especially under co-inocualtion (TD + MET) application, enriching saturated (+ 32%), monounsaturated (+ 13%), and a slight increase in precursor PLFA, and cyclopropyl, while ratios remained stable, relative to control (no PET-MPs; Fig. 6L − Q). Likewise, co-inoculation also increased gram − negative bacteria (+ 31%) and fungal abundance (+ 17%), relative to control (with PET-MPs).

### Multivariate analysis and structural equation model

**Fig. 7:**
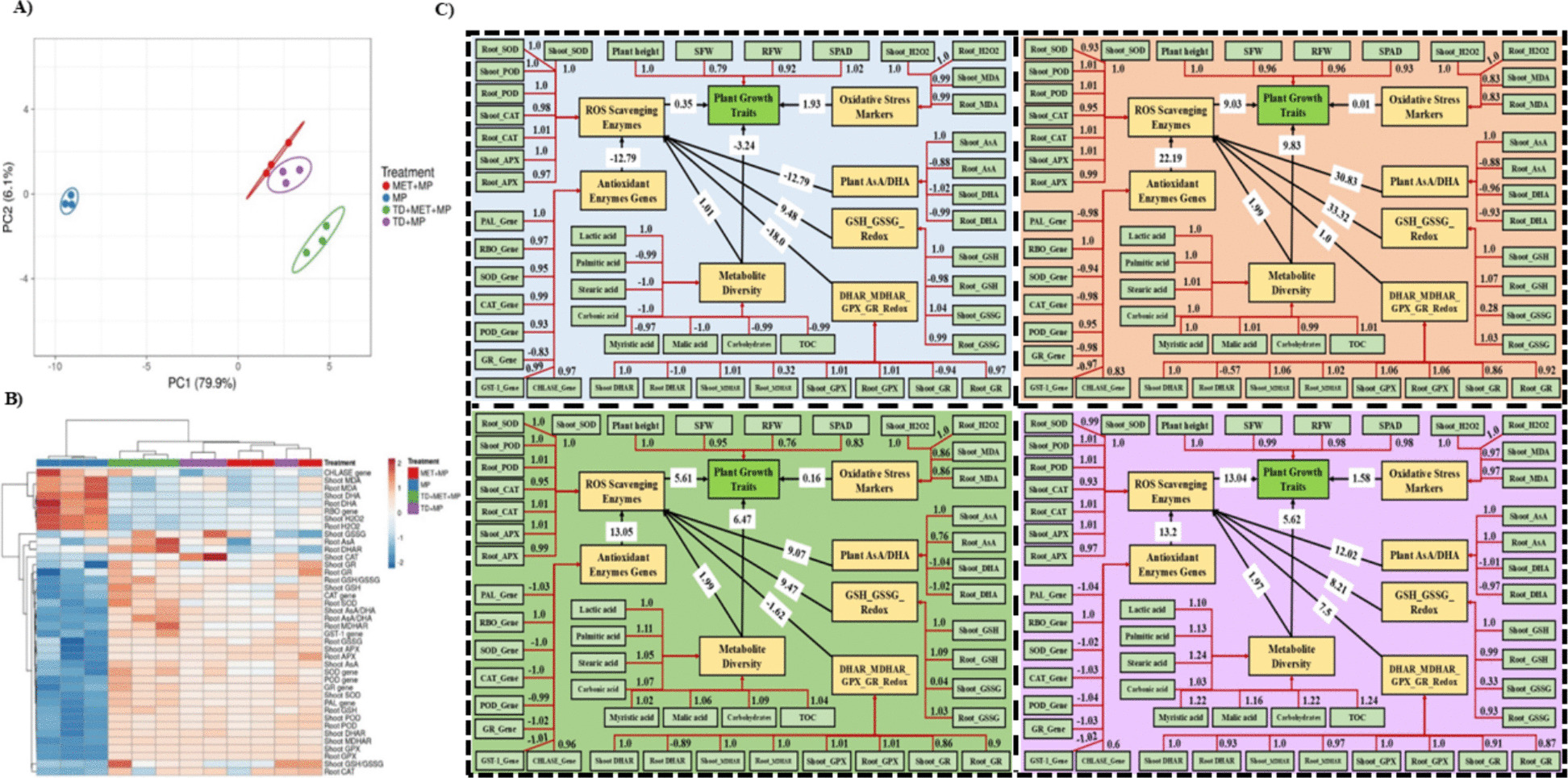
This figure includes (**A**) Principal Component Analysis (PCA) and (**B**) Hierarchical Clustering Analysis (HCA) plots, showing the multivariate analysis of the plant responses, including antioxidant activity and gene expression in maize plants under the influence of fungal inoculation in the presence of PET-MPs stress. **C** Experiment results observed in structural equation model represent the impact of PET-MPs stress (blue block), TD inoculation (orange block), MET inoculation (green block) and co-inoculation (purple block) on maize redox metabolism, the diversity of metabolites in maize and the growth traits observed. Solid arrows show large positive or negative connections between ROS scavenging enzymes, antioxidant genes, redox indices (AsA/DHA, GSH/GSSG), oxidative markers (H₂O₂, MDA) and metabolite outputs (organic acids, sugars, TOC)

PCA analysis indicated that fungal inoculation had a significant effect on plant responses to MP stress, which explained 89% of the variance. PC1 accounted for 79.9%, and PC2 for 6.1%, indicating that fungal inoculation played an important role in causing oxidative stress, antioxidant activity, and gene expression (Fig. 7A). Hierarchical clustering analysis (HCA) showed that some parameters, including oxidative markers, CHLASE and RBO gene, were positively correlated with fungal inoculation while other parameters were negatively correlated with fungal inoculation forming different clusters (Fig. 7B). These results indicate the strong effect of fungal inoculation on the plant response to PET-MPs.

Furthermore, structural equation model showed the correlations between antioxidant capacity, gene expression, AsA/DHA, GSH/GSSG, and oxidative markers (Table S7, Fig. 7 C). PET-MPs stress (blue block) decreased the key plant parameters resulting in lower expression of antioxidant genes, redox modules (AsA/DHA, GSH/GSSG) and scavenging enzymes of ROS, thus, tis led to a significant increase in markers of oxidative stress (+ 1.93). In addition, metabolites such as lactic acid, palmitic acid, and carbohydrates decreased by 0.97 to − 1.01 (Fig. 7C). TD inoculation (orange block) increased expression of antioxidant enzyme genes (22.19), improved redox modules (AsA/DHA + 30.83, GSH/GSSG + 33.32) and increased ROS scavenging enzymes (+ 9.03). This resulted in improvements in oxidative stress markers (0.01) with considerable improvements in plant growth. Moreover, metabolite diversity increased by + 0.99 to + 1.01, suggesting a tradeoff between redox activation and metabolic synthesis (Fig. 7 C). MET inoculation (green block) resulted in mild upregulation of genes involved in antioxidants (13.05), activation of AsA/DHA (+ 9.07), GSH/GSSG (+ 9.47), and other redox modules (+ 3.62). ROS scavenging enzymes increased by + 5.61 and improved plant growth (+ 6.47). Furthermore, the combined TD + MET treatment (purple block) showed the most significant impact in the PET-MPs stressed soil plant system. It upregulated antioxidant genes (13.2) and activated redox modules (AsA/DHA + 12.02, GSH/GSSG + 8.21). ROS scavenging enzymes were greatly enhanced (+ 13.04), reduced oxidative stress markers (− 1.58) and improved plant growth (+ 5.62) (Fig. 7 C). Metabolite diversity increased, with notable improvements in organic acids and TOC (+ 1.10 to + 1.24) were observed. Moreover, structural equation model analysis to soil responses showed that soil enzyme activities were positively correlated with soil nutrients (r^2^ = 0.963), microbial community structure (r^2^ = 0.977), and the PLFA index (r^2^ = 0.576) (Figure S3).

## Discussion

### PET-MPs induced stress and fungal − mediated modulation of maize responses

Exposure to PET-MPs had a severe negative effect on the growth of maize, which was manifested in the decrease of biomass, plant height, and chlorophyll content (SPAD values). These impacts are in line with earlier evidence that MPs affect the soil structure affecting porosity and water uptake and nutrient availability, which limit root growth and overall vegetative growth [62–64]. Moreover, a decrease in chlorophyll content may indicate a decreased ability to absorb nutrients, including nitrogen and magnesium, which are essential in photosynthesis [65]. PET-MPs stress also caused significant oxidative damage, which was observed by elevated concentration of H_2_O_2_ and MDA in roots and shoots. It means that there is an overproduction of reactive oxygen species (ROS) and lipid peroxidation, resulting in cellular damage and dysfunction [66–68]. This oxidative stress mainly influences meristematic tissues thereby suppressing cell division and elongation and eventually plant growth [69–71]. Inoculation with TD and MET as a sole or a combined inoculum reduced this negative impact by enhancing plant physiological functioning and alleviating oxidative injury. Such positive outcomes can be probably linked to the increase in nutrient supply, the development of a better root structure, and the alteration of phytohormonal equilibrium [72, 73]. In addition, TD is also associated with the activation of plant defense systems and antioxidant systems, and MET can produce bioactive compounds like destruxins that may control plant stress responses and limit the damage to cells [21, 74].

The multivariate analyses (PCA and hierarchical clustering) further confirmed that the inoculation by fungi had a significant impact on the plant responses in PET-MPs stress. Clear clustering trends revealed that there was an association between PET-MPs exposure and oxidative stress markers and stress − related gene expression, but fungal treatments diverted plant response to increased antioxidant activity and better redox balance. These results indicate that fungal inoculation is not only stress alleviating but also reprograms physiological and biochemical processes in plants. Additionally, structural equation model showed that PET-MPs stress impairs redox homeostasis by downregulating expression of antioxidant genes and destabilizing major redox systems including AsA/DHA and GSH/GSSG. Conversely, fungal inoculation, especially TD, increased the level of antioxidant genes, ROS scavenging ability, and the maintenance of metabolic homeostasis. The synergistic effects of TD and MET were the most efficient, which implies that the synergistic interaction between the two improves redox regulation and stress resistance. This synergy likely arises from complementary functional traits of the two fungi, enabling more efficient coordination of plant defense and metabolic processes.

### Antioxidant enzyme and gene responses reveal fungal mitigation of PET-MP-induced oxidative stress

The enzymatic transformations under MPs stress imply that they disturb redox balance via stimulation of overproduction of ROS, which is a typical response of plants to abiotic stress. ROS like superoxide radicals, hydrogen peroxide and hydroxyl radicals were able to induce lipid peroxidation, denaturation of proteins and DNA damage if left un − scavenged [75]. The upregulation of antioxidant enzymes is one of the compensatory mechanisms aimed at protecting the cell structures from damage and reconstituting homeostasis of redox process [76].

MPs are previously known to physically interact with the rhizosphere, disturbing root permeability, interfering with nutrient uptake, as well as changing water relations, which can induce ROS overproduction [77]. Moreover, the toxic additives can leach out by MPs or MPs can absorb heavy metals and organic pollutants from the soil which only increases the stress load for the plants [78–80]. The enhancement in antioxidant activity in the current study is coherent with the previous reports implying that plants switch on enzymatic defence systems; it is their first line of defense against the oxidative stress induced by MPs [67, 81].

On the transcriptional level, MPs highly upregulated the expressions of antioxidant and stress − responsive genes including SOD, CAT, POD, and GST^−1^, which testifies to the transcriptional reprogramming that targets ROS detoxification capacity enhancement (Fig. 2I − N). The increased expression of RBO genes indicates increased ROS formation at the plasma membrane level, which could play the role of an early signaling tandem for subsequent activation of defense mechanisms [82, 83]. However, overexpression of the RBO may cause excessive accumulation of ROS, which then may require simultaneous induction of scavenging systems. Moreover, increased PAL and CHLASE suggested that MPs have induced phenylpropanoid metabolism and chlorophyll degradation (likely as a part of a broader stress adaptation mechanism), as suggested by [84] and [85]. Interestingly, reduction of GR indicates a potential failure of glutathione − ascorbate cycle, loss of cell’s ability to regenerate reduced glutathione in which itself plays important role in detoxification of ROS [86–88].

The enzymatic and gene level responses to MPs stress were significantly altered by the fungal inoculations with TD and MET (Fig. 2A − P). Beneficial fungi are well known for their ability to develop symbiotic associations with plant roots, pointing to increased stress resilience via many physiological and molecular mechanisms [89, 90]. In the current study, fungal inoculation significantly enhanced the activity of the antioxidant enzymes as well as modification of gene expression profiles that are indicative of priming maize antioxidant machinery.

The ability of TD species to elicit oxidative stress and systematic resistance is well documented. They do that by generating small molecules like the peptaibols and secondary metabolites that trigger mitogen − activated protein kinase (MAPK) signaling cascades, resulting in the transcription of the defense genes [91]. Furthermore, TD enhances water and nutrient uptake as it improves the colonization of roots which regulates oxidative stress by sustaining cellular metabolism in adverse conditions [72, 92]. The observed decrease in expression of the RBO and CHLASE genes in TD − treated plants implies mitigation of the stress signals and maintenance of chlorophyll content, respectively − an aspect of enhanced physiological homeostasis. Similarly, the MET species act as entomopathogenic fungi with known endophytic capabilities that can also regulate the phytohormone levels, increase antioxidant activity, and stimulate secondary metabolite pathways to improve host plant tolerance to abiotic stresses [93–95]. MET inoculation in this study induced the overexpressed genes of important antioxidants, including SOD, CAT, POD, and GR, thus suggesting its role in strengthening ROS − detoxification mechanisms. The decrease in RBO expression implies the inhibition of the ROS bursts that are induced under stress; this may reduce the cellular damage under MPs stress.

The combination of TD and MET appeared as the most effective treatment, boosting both antioxidant enzyme activity and defense − related genes expression − highlighting complementary and synergistic interaction and reinforcing the adaptive response of the plant in the stressed condition. Such synergism could be due to complementary functional traits − TD is best at rhizosphere colonization and defense priming; MET could favor endophytic interactions and secondary metabolism. Together, they could coactivate several signaling pathways causing increased transcriptional responses and enzymatic defenses. The increased levels of GR, PAL, and GST ^− 1^ in co-inoculated plants indicate the strengthened detoxication system, whereas the reduction of expression of RBO and CHLASE indicates the reduced oxidative load and preservation of the photosynthetic machinery (Fig. 2I − P). Although enzymatic and transcriptional responses helped bring insight into the ROS scavenging, it was also necessary to examine more redox status quo, especially the AsA/DHA system, which is at the center of redox homeostasis.

### Fungal Inoculation Mitigates PET-MPs-induced Redox and Metabolic Disruption in Maize

PET-MPs exposure disrupts redox homeostasis in maize by altering non − enzymatic and enzymatic antioxidant systems. MPs have been reported to disrupt soil structure, nutrient cycling and plant − microbe interactions and eventually, plant physiological balance [77, 96]. In this study, PET-MPs stress had a strong negative impact on AsA − GSH cycle, which was evidenced by low levels of AsA and high levels of DHA in shoots and roots, resulting in a low ratio of AsA/DHA (Fig. 3A–F). This change indicates decreased antioxidative ability and high vulnerability to oxidative injuries, consistent with previous findings under MPs stress [97, 98]. The decrease in AsA regeneration indicates a loss of activity of some of the major recycling enzymes like DHAR and MDHAR, as did other abiotic stresses [99–102].

MP − induced oxidative stress also impacted on the glutathione metabolism which was shown by low level of GSH in the shoots and the change in GSH/GSSG ratio which indicates the impaired redox buffering ability and impaired detoxification of ROS (Fig. 3G–L). There were tissue − specific responses, where roots exhibited comparatively greater GSH accumulation, presumably representing localized defense against direct exposure of MP in the rhizosphere. Simultaneously, the activity of antioxidant enzymes (DHAR, MDHAR, GPX and GR) was also selectively regulated, which is another evidence of redox homeostasis disruption. As an example, the GR activity rose in shoot and reduced in root, indicating a compensatory mechanism in aerial tissues and direct inhibition of roots by MP toxicity [103, 104]. This type of spatial difference in the antioxidant responses has also been observed in other plant systems treated with MPs [105]. In addition to redox regulation, hormonal balance and primary metabolism were also disrupted by PET-MPs (Fig. 4I–V). The reduction of auxins (IAA and IBA) implies the suppression of growth regulating pathways, whereas higher levels of ABA are evidence of stress signaling activation. Simultaneous decrease in SA implies inhibition of defense − related signaling. These hormonal disturbances are in line with the reports that MPs disrupt phytohormone homeostasis and metabolic co − ordination in response to stress [106]. The amino acid buildup also suggests metabolic reprogramming to stress adaptation since the compounds act as osmoprotectants and antioxidants.

The fungal inoculation markedly reduced the oxidative stress caused by PET-MPs by restoring antioxidant metabolite pools and enzymatic activity. TD significantly increased AsA levels and decreased DHA levels, and thus increased AsA/DHA ratio, potentially by stimulating DHAR and MDHAR and enhanced nutrient uptake (e.g., sulfur and iron) to facilitate glutathione and AsA biosynthesis [107–109]. TD has also been reported to activate ROS − scavenging mechanisms and cause systemic resistance, which leads to increased redox stability [110, 111]. In the same manner, MET enhanced redox homeostasis through elevation of AsA levels and stress − responsive signaling pathways, potentially through root colonization and activation of hormone − mediated defense signaling [95, 112–114]. Combined fungal inoculation significantly increased GSH levels and GSH/GSSG ratios, which implies the biosynthesis and recycling of glutathione by the activation of baseline enzymes, including glutathione reductase and γ − glutamylcysteine synthetase. Remarkably, co-inoculation showed the strongest significant effect, demonstrating a synergestic enhancement of antioxidant defense activity. This combined inoculation restores AsA and GSH levels, enhanced antioxidant enzymes (DHAR, MDHAR, GPX, GR) and reinstated effective redox cycling in both tissues (Fig. 4A–H). The complementary mechanism of TD (nutrient mobilization and hormonal control) and MET (stress signaling and endophytic interaction) led to a better ROS detoxification and stabilization of cellular metabolism.

### Microbial inoculants mitigate MPs − induced metabolic dysregulation and restore rhizodeposition

MPs contamination significantly disrupted the metabolic landscape of maize root exudates primarily by affecting central carbon metabolism and modifying sugar, polyol and secondary metabolites profiles (Fig. 5A). The enhanced accumulation of mannitol − talose, fructose, cellobiose and melibiose as well as the pronounced decrease in sucrose, myo − inositol, and raffinose points to the shift in carbon flux and osmolyte distribution into stress under conditions of MP − induced stress. This metabolic switch probably entails a compensatory Osmo − protective response because polyol like mannitol is also accumulated under abiotic stress conditions to scavenge ROS and maintain cellular homeostasis [115, 116]. Moreover, the reduction in sucrose and raffinose indicates disrupted phloem loading and energy transfer that usually resulted from root damage and oxidative stress caused by abiotic stress [117].

MPs also compromised crucial steps of glycolysis and tricarboxylic acid (TCA) cycle as indicated by loss of intermediates like glucose − 1 − phosphate, fructose − 6 − phosphate, 3 − phosphoglycerate, pyruvate, citric acid and oxaloacetate (Fig. 5A). These reductions reflect decreased primary metabolism and energy production that could be a result of oxidative damage to enzymes and impaired mitochondrial functionality [118]. Such metabolic suppression may compromise the generation of ATP, which will influence plant growth and biosynthetic pathways. In addition, stress − induced energy crisis tends to cause rerouting of metabolic flux to stress defense and detoxification instead of growth − promoting processes [118].

In addition, changes in the MP exposure influenced secondary metabolite levels where compounds such as oxalic acid, alanine, stearic acid, and linoleic acid were increased while the beneficial stress − related metabolites like ethanolamine, linolenic acid, shikimic acid, and benzoic acid were decreased (Fig. 5B − I). These changes are indicative of the disruption of the homeostasis in redox and the membrane lipid remodeling. For instance, overaccumulation of oxalic acid may lead to the degradation of cell wall and extraction of calcium under stress [119], whereas decreased linolenic acid and shikimic acid could inhibit production of jasmonate and phenylpropanoids pathways, reducing plant defense mechanisms [120, 121]. However, functional implications of the changed root exudates under MP stress are still poorly understood. Although root exudates are critical in plant growth and microbial interactions it remains unclear how plant root exudates respond to MPs. Studies have indicated that MP disturbs the rhizosphere, influenced pattern of root exudation pattern and microbial communities in rhizosphere [122, 123]. For instance, it has been demonstrated that biodegradable PBAT MPs caused changes in root exudates and microbial composition which affects plant growth. However, the impact of these changes on the plant health and ecosystems needs further investigation to unravel how the interactions of microbes can help to mitigate the stress induced by MPs [123].

Interestingly, microbial inoculation TD or MET, either alone or in combination significantly recovered the metabolite patterns towards homeostasis (Fig. 5A). Inoculated treatments showed that the sucrose and myo − inositol contents were raised but the stress − induced polyols lowered, indicating the recovery of carbohydrate transport and osmotic balance (Fig. 5A). It is known that mycorrhizal fungi and entomopathogenic fungi affect the metabolism of the host’s sugar by increasing the sink strength and stabilizing mechanisms of phloem loading at stress [93, 124–126]. Furthermore, upregulated glycolytic and TCA intermediate products after fungal inoculation indicate better cellular respiration and energy regeneration, which are key in recovery of the plant, and its metabolic resilience [127].

Secondary metabolites were also realigned through fungal treatments (Fig. 5B − I). Lowering oxalic acid and saturated fatty acids such as stearic and palmitic acids imply alleviation of oxidative stress and recovery of membrane stability. Simultaneous rise in the level of linolenic acid, ethanolamine, and shikimic acid might indicate an improvement in the synthesis of stress − protective lipids and secondary metabolites, strengthening the cellular defense. Such adjustments are likely coupled via microbe − controlled reprogramming of metabolic − hormonal signaling pathways including increases in auxin and jasmonate pathways that regulate primary and secondary metabolism in response to abiotic stress, which is well − documented by [128] and [129].

Moreover, changed metabolic patterns were further confirmed by the targeted profiling of root exudates (Fig. 5B − I). Under MP stress, there was a marked increase in lactic acid, a measure of anaerobic respiration and stress − induced fermentation and dramatic drop in total carbohydrates and organic carbon representing the impaired allocation of photosynthates and rhizodeposition. This indicates that microbial symbiosis re − program root metabolism and exudation, enhancing nutrient availability in the rhizosphere and strengthening beneficial microbial associations [130].

### Balancing soil nutrient dynamics and microbial ecology under MPs stress through fungal inoculation

Microplastic pollution has become one of the primary stressors that affect soil biochemistry and microbial ecology, providing ambiguous and often contradictory impacts on enzyme activities and nutrient processes. In the present study, the addition of MP had a notable impact on the chemical properties of soil (DOC, NH_4_^+^, NO_3_^−^, MBC, and MBN), which is consistent with the results reported previously that MPs cause short − to medium − term nutrient pool disturbances in soil [131–133]. Such changes are not only a result of physicochemical interactions of MPs with soil constituents, but also alterations in microbial community structure and functionality, which are reflected by PLFA analysis (Fig. 6).

The contrasting effect of soil enzymes upon MP stress, that is, decrease in urease and amylase activity and increase in sucrase, can be attributed to the action of different microbial functionality strategies induced by varying carbon availability and microbial communities. MPs enhanced the content of DOC, which was presumably due to the creation of sorption surfaces of organic matter and the localized presence of microbial activity [134]. This high DOC selects those microbes with rapid carbon utilization, and this is underpinned by the fact that total bacterial PLFAs, the gram − negative bacteria, and actinomycetes were increased. This sort of copiotrophic microbial group selectively invests in enzymes such as sucrase that can break down easily accessible carbon (e.g., sucrose), which explains why sucrase activity was induced in response to MP stress.

Conversely, there was a decrease in urease and amylase activities with the addition of MP. Nitrogen mineralization is closely associated with urease which is mainly produced by microbial metabolism and root exudation. This decrease in urease activity is probably due to physical restraints of microbial access to nutrients, enzyme adsorption over the MP surfaces, and taxonomic switching away of nitrogen − mineralizing microbes. Likewise, amylase acting upon large polysaccharides like starch can be inhibited by the microbial preference towards the simpler carbon sources in MP − enriched DOC reducing the starch metabolic requirement.

This functional interpretation is also backed by the PLFA − based pointers. The rise in saturated/monounsaturated and G +/G − ratios in addition to a decline in the fungi/bacteria ratio suggest a microbial community in nutritional imbalance and adverse conditions, with decrease in fungal contributions to soil functioning. Fungi also play an important role in extracellular enzyme production in cycling of complex carbon and nitrogen; therefore, their relative loss in MP stress assists in explaining the general loss of hydrolases like urease and amylase. In the meantime, the reduction in cy/pre PLFA ratios indicates less carbon constraint, and a higher rate of bacterial growth, which goes in line with high sucrase activity and bacterial domination.

These imbalances caused by MPs were alleviated with fungal inoculation, especially the combined TD and MET treatment as they reorganized the microbial community and regained functional capacity. Addition of fungi increased the availability of NH_4_^+^, and NO_3_, which would have been possible by increasing the mineralization of organic nitrogen and microbial interactions. In line with this, the activity of hydrolases and oxidoreductases (urease, dehydrogenase, phosphatase and catalase) improved significantly after fungal inoculation with indicators of better nutrient cycling and alleviation of stress. The decrease in sucrase activity following fungal inoculation indicates that the carbon acquisition mode is less dependent on stress to more balanced carbon usage that is in line with an increase in amylase activity and a decrease in reliance on sucrose hydrolysis.

Overall, the combination of enzyme activity patterns with PLFA − based microbial community changes allows concluding that MPs are the main cause of a microbial system dominating by bacteria and adapted to stress and labile carbon consumption at the cost of nitrogen and complex carbon cycles. Inoculation systems with fungi negate these effects by restoring fungal mechanisms of decomposition, enhancing mineralization of nutrients, and maintaining the biochemical processes of the soil when stressed by MP. This integrated functional–ecological perspective provides a robust explanation for the seemingly contradictory enzyme responses observed in MP − contaminated soils.

Moreover, there are certain limitations to this study as it directly measures degradation of PET − MPs by the fungal isolates and no quantification of PET-MPs in the soil or plant tissues was conducted; therefore, the findings are indicative of only stress reduction. Furthermore, the experiment was carried out in controlled conditions at relatively high MP concentrations, which are not necessarily representative of field conditions. Also, the persistence of fungus, contact with native microbiota, and possible physicochemical alteration of PET-MPs were not assessed. Future research needs to be directed at the direct assessment of fungal strains ability to degrade PET-MPs. To quantify PET-MPs remnants in soil and plant tissues in order to determine their destiny and possible movement in soil and plant system. Also, the incorporation of omics − based methods may offer further understanding of the molecular pathways involved in the interaction of plants, fungi, and microplastic. To evaluate the potential practical applicability and consistency of fungal inoculation strategies in real − world agricultural environments, field − scale testing will be required over a variety of soil types and environmental conditions.

## Conclusion

This study shows that inoculation of fungal inoculation (Trichoderma and Metarhizium), either solely or in combination, significantly mitigate the detrimental impact of MPs stress on maize plants. Fungal treatments were effective in the recovery of redox balance and antioxidant enzyme activity in addition to the reprogramming of primary metabolic pathways disturbed by MP − induced stress. Moreover, our results also reveal the huge effect of fungal inoculation on soil health in terms of enhanced carbon and nitrogen cycling, enhanced enzyme activities, and higher microbial community diversity especially in the MP − contaminated soils. The combination of TD and MET worked the best to promote plant growth and promote beneficial soil characteristics. These findings highlight the possibilities of fungal consortia as one of the possible integrated solution to the problems posed by plastic pollution in agricultural systems. By enhancing resilience of plant and soil functionality, the approach represents a viable solution to sustainable agriculture in MP affected ecosystems. Prospective research needs to address the scalability of these fungal inoculants in the real − field situation to certify them as feasible strategies to reduce plastic − induced stress in the long − term.

## Supplementary Information

Supplementary Material 1.

## Data Availability

The data supporting the reported results of this article will be made available by the authors on request.

## Abbreviations

AsA: Ascorbic acid
AsA − GSH: Ascorbate − glutathione
B: Boron
CAT: Catalase
CFI: Comparative fit index
CHLASE: Chlorophyllase
DHA: Dehydroascorbic acid
DHAR: Dehydroascorbate reductase
DOC: Dissolved organic carbon
DTT: Dithiothreitol
EC: Electrical conductivity
FID: Flame ionization detector
FIV − G: Fungal Infections and Virology Group
GC: Gas chromatography
GC − MS: Gas chromatography − mass spectrometry
GHGs: Greenhouse gas emissions
GPX: Glutathione peroxidase
GR: Glutathione reductase
GSH: Reduced glutathione
GSSG: Oxidized glutathione
H₂O₂: Hydrogen peroxide
IESE: Institute of Environmental Sciences and Engineering
IS1: Internal standard 1
K: Potassium
KI: Potassium iodide
LDPE: Low − density polyethylene
MDA: Malondialdehyde
MBC: Microbial biomass carbon
MDHAR: Mono − dehydroascorbate reductase
MBN: Microbial biomass nitrogen
MET: Metarhizium
MPs: Microplastics
MSTFA: N − methyl − N − (trimethylsilyl) trifluoroacetamide
NARC: National Agriculture Research Center
NUST: National University of Sciences and Technology
P: Phosphorus
PCA: Principal component analysis
PDA: Potato dextrose agar
PET: Polyethylene terephthalate
PBS: Phosphate buffered saline
PLFAs: Phospholipid fatty acids
PNP: P − Nitrophenol
POD: Peroxidase
qRT − PCR: Quantitative real − time polymerase chain reaction
RBO: Respiratory burst oxidase
RMSEA: Root − mean − square error of approximation
ROS: Reactive oxygen species
SCEE: School of Civil and Environmental Engineering
SDW: Shoot dry weight
SE: Standard error
SEM: Scanning electron microscope
SOD: Superoxide dismutase
TCA: Trichloroacetic acid
TD: Trichoderma
TDMET: Trichoderma + Metarhizium
TOC: Total organic carbon
X^2^: Chi − square
