# Supplementary material for: Synergistic fungal consortia enhance maize growth and soil biological functions under microplastic stress
Source: BMC Plant Biol. 2026 May 20;26:1191. doi: 10.1186/s12870-026-08924-w (PMC13366829; doi:10.1186/s12870-026-08924-w)
Supplement: Supplementary file 1 — Supplementary Material 1. [file 12870_2026_8924_MOESM1_ESM.docx]

**
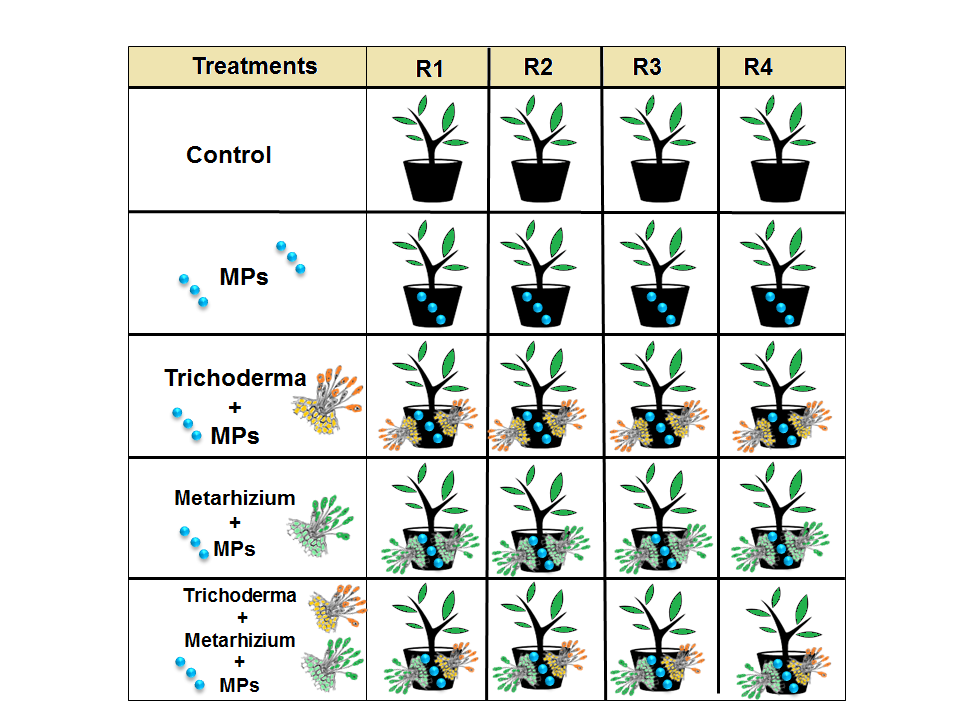
**

**Figure S1:** Pictorial representation of experimental treatments used in the study.

*
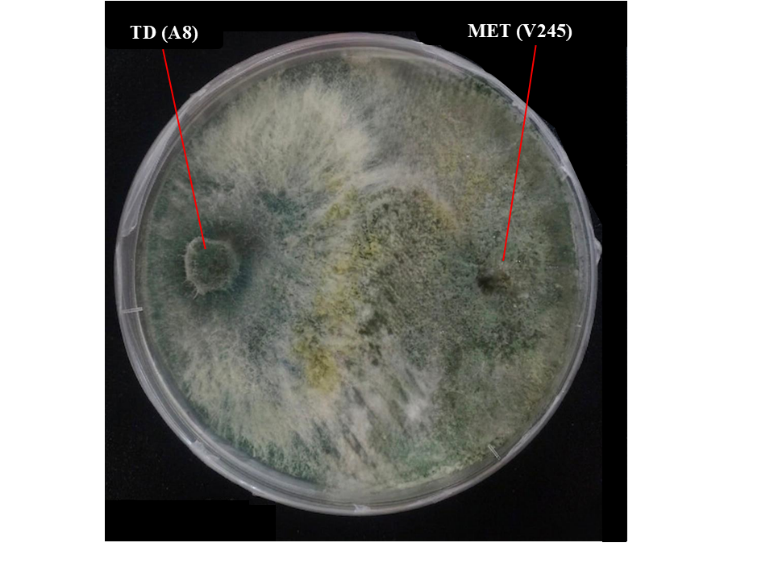
*

**Figure S2:** Synergistic interaction between Trichoderma and Metarhizium on PDA plate.

**
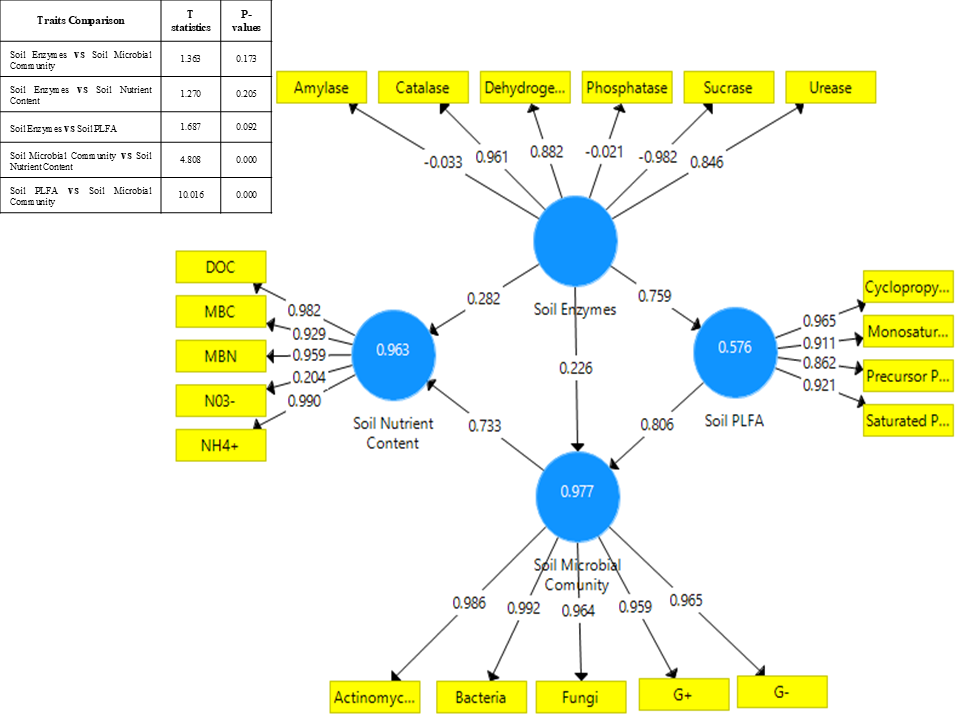
**

**Figure S3:** Structural equation model (SEM) was derived to predict the positive and negative influence of each attribute. Significance levels at ***(p ≤ 0.001), ** (p ≤ 0.01), and *(p ≤ 0.05) show very strong, strong, and slightly positive or negative impacts, respectively.

**Table S1:** This table provides a comprehensive list of the primer sequences used in the study to check the relative expression of antioxidant enzymes.

| **Genes** | **Forward Primer** | **Reverse primer** | **Amplicon size (bp)** |
| --- | --- | --- | --- |
| **Actin** | 5’ CTTGCACCTAGCAGCATGAA 3’ | 5’ GGACAATGGATGGACCTGAC 3’ | 148 |
| **RBO** | 5′ACGGGGTGTGATAGAGATGC 3′ | 5′TTTTTCCAGTTGGGTCTTGC 3′ | 160 |
| **SOD** | 5’GGTTTCCATGTCCATGCTCT 3’ | 5’ATTGTGAAGGTGGCAGTTCC 3’ | 152 |
| **CAT** | 5’TCAGCTGCCAGTTAATGCAC 3’ | 5’GACAGCAGGTGGAGTTGGAT 3’ | 158 |
| **POD** | 5'TTCGAACGGAAAAAGATGCT 3' | 5'AACCCTCCATGAAGGACCTC 3' | 136 |
| **GR** | 5'AAGGCAAAAGAAGGTGCTGA 3' | 5'AGTTCCCTTGCTGGTCTTCA 3' | 166 |
| **GST-1** | 5'CGTCGTCGAAGAAGAAGAGG 3' | 5'TTTTTGGTGGGAGTTCCAAG 3' | 170 |
| **CHLASE** | 5’GAATATCCGGTGGTGATGCT 3’ | 5’TCCGCCGTTGATTTTATCTC 3' | 162 |
| **PAL** | 5’AAACTCCGTCAACGACAACC3’ | 5’AGCGAACATGAGCTTCCCTA3’ | 185 |

**Table S2:** The table presents mean ± SE for growth, oxidative stress, antioxidant activity, gene expression, and soil parameters under various treatments.

| **Treatment** | **Control** | **MMP** | **MP** | **TMMP** | **TMP** |
| --- | --- | --- | --- | --- | --- |
| **Plant height** | 27.66 ± 1.45 | 43 ± 0.57 | 18.66 ± 0.88 | 54.66 ± 1.76 | 48 ± 0.57 |
| **SDW** | 1.96 ± 0.10 | 1.71 ± 0.18 | 1.3 ± 0.01 | 2.7 ± 0.14 | 2.12 ± 0.1 |
| **RDW** | 0.65 ± 0.03 | 0.57 ± 0.06 | 0.43 ± 0.01 | 0.9 ± 0.04 | 0.7 ± 0.03 |
| **SPAD** | 36.33 ± 0.88 | 28.16 ± 1.64 | 22.66 ± 1.45 | 40.33 ± 1.45 | 32.46 ± 1.51 |
| **Shoot H2O2** | 140.14 ± 3.29 | 297.96 ± 5.84 | 432.83 ± 8.09 | 233.25 ± 6.11 | 268.09 ± 8.07 |
| **Root H2O2** | 68.98 ± 1.64 | 147.89 ± 2.92 | 215.32 ± 4.04 | 115.54 ± 3.05 | 132.96 ± 4.03 |
| **Shoot MDA** | 42.37 ± 1.29 | 56.28 ± 3.75 | 68.91± 1.66 | 51.69 ± 1.08 | 58.48 ± 2.65 |
| **Root MDA** | 19.11 ± 0.61 | 25.73 ± 1.78 | 31.75 ± 0.79 | 23.55 ± 0.51 | 26.78± 1.26 |
| **Shoot SOD** | 87.50 ± 4.13 | 220.02± 5.19 | 159.91 ± 4.95 | 239.25 ± 5.06 | 216.65 ± 6.11 |
| **Root SOD** | 49.48 ± 2.15 | 99.69 ± 1.71 | 77.24 ± 1.14 | 124.25 ± 5.06 | 101.65 ± 6.11 |
| **Shoot POD** | 264.07 ± 6.61 | 463.51± 8.32 | 370.52 ± 3.70 | 567.46 ± 3.51 | 468.62 ± 5.34 |
| **Root POD** | 224.40 ± 4.19 | 483.84 ± 3.58 | 387.52± 2.20 | 566.46 ± 2.91 | 525.62 ± 5.34 |
| **Shoot CAT** | 0.57 ± 0.01 | 1.20 ± 0.05 | 0.90 ± 0.03 | 1.5 ± 0.11 | 1.28 ± 0.05 |
| **Root CAT** | 0.50 ± 0.01 | 1.53 ± 0.032 | 0.86 ± 0.01 | 1.83 ± 0.02 | 1.45 ± 0.02 |
| **Shoot APX** | 0.89 ± 0.05 | 2.99 ± 0.01 | 1.77 ± 0.02 | 3.89 ± 0.01 | 2.93 ± 0.02 |
| **Root APX** | 0.55 ± 0.03 | 1.09 ± 0.01 | 0.82 ± 0.03 | 1.30± 0.06 | 1.13 ± 0.02 |
| **RBO gene** | 1.01 ± 0.12 | 2.43 ± 0.14 | 3.66 ± 0.14 | 1.95 ± 0.01 | 2.02 ± 0.04 |
| **SOD gene** | 1.02 ± 0.17 | 4.13 ± 0.09 | 2.63 ± 0.17 | 5.06 ± 0.14 | 4.63 ± 0.17 |
| **CAT gene** | 1.0 ± 0.05 | 4.66 ± 0.14 | 3.23± 0.14 | 5.43 ± 0.14 | 5.13 ± 0.20 |
| **POD gene** | 1.0 ± 0.0 | 1.98 ± 0.01 | 1.43 ± 0.08 | 3.01 ± 0.07 | 2.19 ± 0.12 |
| **GR gene** | 1.0 ± 0.01 | 2.06 ± 0.06 | 0.56 ± 0.14 | 2.51 ± 0.11 | 2.16 ± 0.12 |
| **GST-1 gene** | 1.01 ± 0.02 | 3.02 ± 0.03 | 2.06 ± 0.06 | 3.43 ± 0.08 | 3.13 ± 0.09 |
| **CHLASE gene** | 1.03 ± 0.05 | 2.03 ± 0.035 | 2.73 ± 0.14 | 2.46 ± 0.12 | 2.03 ± 0.14 |
| **PAL gene** | 1.02 ± 0.04 | 4.13 ± 0.09 | 1.93 ± 0.11 | 4.66 ± 0.08 | 4.22 ± 0.15 |
| **Shoot AsA** | 0.94 ± 0.01 | 0.73 ± 0.03 | 0.43 ± 0.02 | 0.82 ± 0.01 | 0.77 ± 0.02 |
| **Root AsA** | 0.38 ± 0.06 | 0.49 ± 0.01 | 0.44 ± 0.02 | 0.66 ± 0.02 | 0.556 ± 0.02 |
| **Shoot DHA** | 0.217 ± 0.01 | 0.47 ± 0.02 | 0.91 ± 0.02 | 0.431 ± 0.02 | 0.46 ± 0.02 |
| **Root DHA** | 1.17 ± 0.06 | 1.34± 0.06 | 2.49 ± 0.13 | 1.32 ± 0.05 | 1.45 ± 0.05 |
| **Shoot AsA/DHA** | 4.34 ± 0.19 | 1.57± 0.09 | 0.47 ± 0.03 | 1.9 ± 0.08 | 1.68 ± 0.05 |
| **Root AsA/DHA** | 0.32 ± 0.01 | 0.33 ± 0.02 | 0.17 ± 0.01 | 0.40 ± 0.02 | 0.34 ± 0.02 |
| **Shoot GSH** | 0.55 ± 0.01 | 0.51 ± 0.01 | 0.41± 0.01 | 0.58 ± 0.02 | 0.55 ± 0.01 |
| **Root GSH** | 0.33 ± 0.01 | 0.66 ± 0.01 | 0.42 ± 0.01 | 0.69 ± 0.01 | 0.65 ± 0.01 |
| **Shoot GSSG** | 0.73 ± 0.01 | 0.33 ± 0.02 | 0.33 ± 0.01 | 0.35 ± 0.02 | 0.35 ± 0.02 |
| **Root GSSG** | 0.40± 0.01 | 0.39 ± 0.01 | 0.313 ± 0.01 | 0.39 ± 0.01 | 0.399 ± 0.01 |
| **Shoot GSH/GSSG** | 0.75 ± 0.032 | 1.55 ± 0.14 | 1.24± 0.01 | 1.68 ± 0.12 | 1.61 ± 0.13 |
| **Root GSH/GSSG** | 0.80 ± 0.02 | 1.71 ± 0.01 | 1.36 ± 0.02 | 1.77 ± 0.03 | 1.63 ± 0.04 |
| **Shoot DHAR** | 274.98 ± 3.67 | 259.23 ± 1.94 | 172.44 ± 5.71 | 266.50 ± 2.70 | 269.11 ± 3.60 |
| **Root DHAR** | 59.23 ± 5.71 | 146.02 ± 1.94 | 161.77 ± 3.67 | 183.51 ± 2.41 | 155.90 ± 3.60 |
| **Shoot MDHAR** | 1.51 ± 0.02 | 1.53 ± 0.01 | 0.98 ± 0.01 | 1.58 ± 0.01 | 1.57 ± 0.01 |
| **Root MDHAR** | 1.5 ± 0.01 | 2.02 ± 0.03 | 1.48 ± 0.021 | 2.36 ± 0.12 | 2.16 ± 0.09 |
| **Shoot GPX** | 727.58 ± 2.24 | 777.73 ± 4.48 | 414.32 ± 6.31 | 771.43 ± 2.44 | 767.67 ± 4.62 |
| **Root GPX** | 913.59 ± 2.66 | 952.73 ± 4.48 | 589.32 ± 6.31 | 946.43 ± 2.44 | 942.67 ± 4.62 |
| **Shoot GR** | 0.32 ± 0.01 | 0.48 ± 0.01 | 0.423 ± 0.01 | 0.48 ± 0.01 | 0.47 ± 0.01 |
| **Root GR** | 0.37 ± 0.01 | 0.30 ± 0.01 | 0.23 ± 0.01 | 0.29 ± 0.01 | 0.29 ± 0.01 |
| **Lactic acid** | 1.23 ± 0.01 | 2.29 ± 0.17 | 2.66 ± 0.03 | 1.43 ± 0.17 | 1.96 ± 0.03 |
| **Palmitic acid** | 3.23 ± 0.14 | 1.76 ± 0.073 | 1.163 ± 0.03 | 2.7 ± 0.11 | 2.13 ± 0.08 |
| **Stearic acid** | 1.82 ± 0.05 | 1.34 ± 0.07 | 0.69 ± 0.02 | 2.01 ± 0.04 | 1.55 ± 0.06 |
| **Carbonic acid** | 3.5 ± 0.11 | 2.97 ± 0.06 | 1.653 ± 0.04 | 3.23± 0.08 | 2.87 ± 0.11 |
| **Myristic acid** | 2.48 ± 0.13 | 2.19 ± 0.15 | 1.23 ± 0.02 | 2.4 ± 0.11 | 2.3 ± 0.11 |
| **Malic acid** | 3.5 ± 0.11 | 1.91 ± 0.04 | 1.36 ± 0.02 | 2.89 ± 0.18 | 2.65 ± 0.11 |
| **Carbohydrates** | 4.66 ± 0.14 | 3.26 ± 0.26 | 2.36 ± 0.13 | 4.16± 0.14 | 3.93 ± 0.12 |
| **Total organic carbon** | 4.0 ± 0.15 | 3.13 ± 0.20 | 1.46 ± 0.12 | 3.73 ± 0.08 | 3.34 ± 0.08 |
| **Dehydrogenase** | 69.69 ± 2.58 | 45.15 ± 3.74 | 145.03 ± 3.80 | 128.22 ± 2.86 | 162.49 ± 3.77 |
| **Phosphatase** | 126.05 ± 3.18 | 78.72 ± 3.08 | 108.26 ± 1.72 | 101.92 ± 4.04 | 117.77 ± 1.86 |
| **Urease** | 110.22 ± 1.90 | 53.47 ± 2.44 | 191.58 ± 3.88 | 186.62 ± 2.62 | 200.14 ± 2.28 |
| **Catalase** | 2.06 ± 0.07 | 2.09 ± 0.11 | 3.05 ± 0.08 | 2.99 ± 0.01 | 3.13 ± 0.09 |
| **Amylase** | 1458.14 ± 4.88 | 373.96 ± 6.87 | 1048.18 ± 25.92 | 963.29 ± 11.07 | 1150.78 ± 24.08 |
| **Sucrase** | 439.22 ± 4.83 | 411.25 ± 3.14 | 311.66 ± 3.40 | 306.86 ± 3.90 | 334.38 ± 7.76 |
| **MBC** | 40.52 ± 1.51 | 84.94 ± 5.16 | 88.70 ± 7.89 | 85.15 ± 5.36 | 93.70 ± 3.66 |
| **MBN** | 11.71 ± 0.20 | 17.40 ± 0.33 | 17.67 ± 0.34 | 17.67 ± 0.33 | 18.34 ± 0.27 |
| **Saturated PLFA** | 0.08 ± 0.00 | 0.18 ± 0.01 | 0.18 ± 0.01 | 0.18 ± 0.01 | 0.24 ± 0.03 |
| **G** | 0.44 ± 0.01 | 0.78 ± 0.01 | 0.79 ± 0.01 | 0.78 ± 0.01 | 0.83 ± 0.02 |
| **G-** | 0.09 ± 0.00 | 0.21 ± 0.00 | 0.27 ± 0.03 | 0.21 ± 0.00 | 0.32 ± 0.01 |
| **Bacteria** | 0.53 ± 0.01 | 0.98 ± 0.01 | 1.06 ± 0.04 | 0.99 ± 0.01 | 1.15 ± 0.01 |
| **Fungi** | 0.12 ± 0.00 | 0.22 ± 0.00 | 0.27 ± 0.01 | 0.26 ± 0.01 | 0.35 ± 0.02 |
| **Actinomycetes** | 0.09 ± 0.00 | 0.22 ± 0.01 | 0.28 ± 0.01 | 0.24 ± 0.02 | 0.30 ± 0.01 |
| **Monosaturated PLFA** | 0.15 ± 0.00 | 0.34 ± 0.03 | 0.35 ± 0.05 | 0.31 ± 0.01 | 0.38 ± 0.04 |
| **Cyclopropyl PLFA** | 0.03 ± 0.00 | 0.05 ± 0.00 | 0.05 ± 0.00 | 0.05 ± 0.00 | 0.05 ± 0.00 |
| **Precursor PLFA** | 0.07 ± 0.00 | 0.18 ± 0.02 | 0.22 ± 0.03 | 0.18 ± 0.01 | 0.21 ± 0.02 |
| **G/G-** | 4.83 ± 0.09 | 3.78 ± 0.06 | 3.01 ± 0.36 | 3.76 ± 0.08 | 2.62 ± 0.11 |
| **Fungi/Bacteria** | 0.23 ± 0.00 | 0.23 ± 0.00 | 0.25 ± 0.00 | 0.26 ± 0.01 | 0.30 ± 0.02 |
| **Saturated PLFA/Monosaturated PLFA** | 0.54 ± 0.01 | 0.54 ± 0.05 | 0.52 ± 0.05 | 0.57 ± 0.03 | 0.65 ± 0.16 |
| **Cyclopropyl PLFA/Precursor PLFA** | 0.34 ± 0.02 | 0.27 ± 0.03 | 0.23 ± 0.04 | 0.26 ± 0.03 | 0.27 ± 0.04 |
| **DOC** | 45.98 ± 1.33 | 68.58 ± 1.92 | 78.23 ± 2.75 | 74.09 ± 1.38 | 77.39 ± 1.38 |
| **NH4** | 4.47 ± 0.15 | 6.30 ± 0.12 | 7.10 ± 0.12 | 7.00 ± 0.12 | 7.50 ± 0.06 |
| **N03-** | 2.91 ± 0.01 | 1.30 ± 0.12 | 3.06 ± 0.07 | 3.16 ± 0.09 | 3.47 ± 0.09 |

**Table S3:** The table displays effect size values (Cohen's d) for various parameters comparing different treatment groups under MP stress. Positive values indicate treatment enhancement, while negative values suggest reduced effects.

| **Parameter** | **Control vs MP** | **MP vs MMP** | **MP vs TMP** | **MP vs TMMP** |
| --- | --- | --- | --- | --- |
| **Plant height** | -7.5 | 13.92 | 18.46 | 16.74 |
| **SDW** | -9.29 | -1.72 | 1.6 | 6.08 |
| **RDW** | -9.84 | -1.69 | 1.67 | 7.07 |
| **SPAD** | -11.4 | -6.21 | -3.13 | 3.34 |
| **Shoot H2O2** | 47.4 | 33.3 | 20.76 | 18.98 |
| **Root H2O2** | 47.46 | 33.32 | 20.8 | 19.01 |
| **Shoot MDA** | 17.85 | 4.96 | 7.73 | 7.83 |
| **Root MDA** | 17.91 | 4.98 | 7.75 | 7.9 |
| **Shoot SOD** | 15.88 | 28.26 | 24.77 | 32.86 |
| **Root SOD** | 16.13 | 25.85 | 11.39 | 19.23 |
| **Shoot POD** | 19.87 | 26.54 | 34.04 | 57.33 |
| **Root POD** | 48.75 | 66.58 | 62.76 | 94.83 |
| **Shoot CAT** | 14.76 | 17.47 | 19.69 | 11.91 |
| **Root CAT** | 36 | 43.45 | 60.08 | 84.12 |
| **Shoot APX** | 23.11 | 58.24 | 53.57 | 83.21 |
| **Root APX** | 9 | 24.15 | 22.75 | 15.81 |
| **RBO gene** | 20.32 | 10.89 | 11.29 | 11.04 |
| **SOD gene** | 9.47 | 22.87 | 21.24 | 25.94 |
| **CAT gene** | 21.21 | 34.82 | 28.33 | 42.14 |
| **POD gene** | 7.6 | 138.59 | 14.02 | 40.61 |
| **GR gene** | -4.43 | 24.64 | 13.62 | 19.33 |
| **GST-1 gene** | 23.48 | 78.84 | 32.52 | 41.5 |
| **CHLASE gene** | 16.17 | 23.17 | 9.51 | 15.56 |
| **PAL gene** | 11 | 44.66 | 29.15 | 57.55 |
| **Shoot AsA** | -32.26 | -9.39 | -10.75 | -12 |
| **Root AsA** | 1.34 | 2.56 | 3.94 | 6.26 |
| **Shoot DHA** | 43.83 | 16 | 15.37 | 13.53 |
| **Root DHA** | 13.04 | 2.83 | 5.07 | 2.72 |
| **Shoot AsA/DHA** | -28.45 | -18.63 | -19.15 | -16.74 |
| **Root AsA/DHA** | -15 | 0.63 | 1.26 | 5.06 |
| **Shoot GSH** | -14 | -4 | 0 | 1.9 |
| **Root GSH** | 9 | 33 | 32 | 36 |
| **Shoot GSSG** | -40 | -25.3 | -24.03 | -24.03 |
| **Root GSSG** | -8.7 | -1 | -0.1 | -1 |
| **Shoot GSH/GSSG** | 20.67 | 7.88 | 9.08 | 10.59 |
| **Root GSH/GSSG** | 28 | 57.55 | 26.25 | 38.05 |
| **Shoot DHAR** | -21.36 | -5.37 | -1.61 | -2.63 |
| **Root DHAR** | 21.36 | 20.35 | 20.25 | 28.36 |
| **Shoot MDHAR** | -33.52 | 1.26 | 3.79 | 4.43 |
| **Root MDHAR** | -1.22 | 23.26 | 10.31 | 10.1 |
| **Shoot GPX** | -66.16 | 14.16 | 11.04 | 18.72 |
| **Root GPX** | -66.97 | 10.62 | 7.71 | 12.87 |
| **Shoot GR** | 10.3 | 16 | 15 | 16 |
| **Root GR** | -14 | -7 | -8 | -8 |
| **Lactic acid** | 63.95 | 8.8 | 32.65 | 1.66 |
| **Palmitic acid** | -20.42 | -13.17 | -9.65 | -4.21 |
| **Stearic acid** | -29.68 | -7.89 | -4.89 | 4.2 |
| **Carbonic acid** | -22.32 | -5.98 | -5.73 | -2.81 |
| **Myristic acid** | -13.44 | -2.07 | -1.49 | -0.66 |
| **Malic acid** | -27.07 | -19.21 | -7.73 | -4.09 |
| **Carbohydrates** | -17.03 | -6.7 | -5.6 | -3.57 |
| **Total organic carbon** | -18.7 | -4.92 | -5.49 | -2.25 |
| **Dehydrogenase** | 4.41 | -15.3 | -14.41 | -18.04 |
| **Phosphatase** | 8.73 | -6.84 | -3.73 | -8.87 |
| **Urease** | 15.01 | -24.62 | -30.4 | -35.88 |
| **Catalase** | -0.19 | -6.06 | -6.92 | -6.11 |
| **Amylase** | 104.97 | -20.53 | -36.93 | -25.33 |
| **Sucrase** | 3.97 | 17.58 | 17.04 | 7.5 |
| **MBC** | -6.75 | -0.33 | -0.02 | -1.13 |
| **MBN** | -12.1 | -0.46 | -0.46 | -1.81 |
| **Saturated PLFA** | -7.31 | 0 | 0.07 | -1.36 |
| **G+** | -20.6 | -0.65 | -0.09 | -2.26 |
| **G-** | -37.32 | -1.66 | -0.52 | -11.83 |
| **Bacteria** | -26.71 | -1.45 | -0.17 | -9.6 |
| **Fungi** | -15.79 | -3.73 | -2.84 | -5.84 |
| **Actinomycetes** | -9.12 | -3.1 | -1.08 | -4 |
| **Monosaturated PLFA** | -5.03 | -0.2 | 0.59 | -0.69 |
| **Cyclopropyl PLFA** | -10.73 | 0.32 | 0.64 | -1.08 |
| **Precursor PLFA** | -5.7 | -0.96 | -0.01 | -0.75 |
| **G/G-** | 7.57 | 1.74 | 0.14 | 7.42 |
| **Fungi/Bacteria** | 0.7 | -8.11 | -3.55 | -3.8 |
| **Saturated PLFA/Monosaturated PLFA** | 0.09 | 0.19 | -0.44 | -0.55 |
| **Cyclopropyl PLFA/Precursor PLFA** | 1.73 | 0.73 | 0.33 | 0.1 |
| **DOC** | -7.9 | -2.35 | -1.9 | -3.04 |
| **NH_4_^+^** | -8.07 | -4 | -3.5 | -7.59 |
| **N0_3_^-^** | 11.37 | -10.53 | -10.33 | -12.18 |

**Table S4:** The table summarizes the effects of the treatments under microplastic (MP) stress on growth, oxidative stress, antioxidant gene expressions, and soil properties. Independent and combined effects of TD and MET are compared, and the interaction between TD and MET is estimated using Bliss Independence model, which shows the independent and synergistic effects of TD and MET.

| **Feature** | **MP Stress (No Treatment)** | **TD+MP (%)** | **MET+MP (%)** | **TD+MET+MP (%)** | **Synergistic Expected Combined Effect (%)** | **Synergistic Observed Combined Effect (%)** |
| --- | --- | --- | --- | --- | --- | --- |
| **Growth and Oxidative Stress Markers** | | | | | | |
| Plant Height | -33 | 157 | 130 | 193 | 82.9 | 193 |
| Shoot Fresh Weight | -22 | 55 | 34 | 93 | 70.3 | 93 |
| Root Fresh Weight | -33 | 62 | 31 | 107 | 73.78 | 107 |
| SPAD | -38 | 43 | 24 | 78 | 56.68 | 78 |
| H2O2 (Shoot) | 209 | -38 | -31 | -46 | -80.78 | -46 |
| H2O2 (Root) | 213 | -38 | -31 | -46 | -80.78 | -46 |
| MDA (Shoot) | 63 | -15 | -18 | -25 | -35.7 | -25 |
| MDA (Root) | 66 | -16 | -19 | -26 | -38.04 | -26 |
| SOD (Shoot) | 83 | 35 | 38 | 50 | 59.7 | 50 |
| POD (Shoot) | 40 | 26 | 25 | 53 | 44.5 | 53 |
| CAT (Shoot) | 57 | 42 | 33 | 65 | 61.14 | 65 |
| APX (Shoot) | 98 | 64 | 68 | 118 | 88.47 | 118 |
| SOD (Root) | 56 | 32 | 29 | 168 | 51.72 | 168 |
| POD (Root) | 73 | 36 | 25 | 46 | 52 | 46 |
| CAT (Root) | 70 | 69 | 78 | 113 | 93.18 | 113 |
| APX (Root) | 43 | 38 | 59 | 59 | 74.58 | 59 |
| **Antioxidant Gene Expressions** | | | | | | |
| SOD | 163 | 76 | 57 | 92 | 77.03 | 68 |
| POD | 43 | 53 | 38 | 109 | 74.56 | 341 |
| CAT | 223 | 59 | 44 | 68 | 70.86 | 109 |
| GR | -43 | 281 | 264 | 341 | -120.52 | -53 |
| GST-1 | 106 | 52 | 47 | 66 | -94.3 | -47 |
| PAL | 93 | 119 | 114 | 142 | -80.18 | -10 |
| RBO | 267 | -45 | -34 | -47 | 89.68 | 92 |
| CHLASE | 173 | -26 | -26 | -10 | 97.34 | 142 |
| **Ascorbate and Dehydroascorbate Metrics** | | | | | | |
| AsA (Shoot) | -54 | 81 | 71 | 91 | -107.32 | -47 |
| AsA (Root) | 16 | 24 | 2 | 21 | 111.88 | 128 |
| DHA (Shoot) | 319 | -49 | -48 | -53 | -104.6 | 305 |
| DHA (Root) | 112 | -42 | -46 | -47 | 118.44 | 305 |
| AsA/DHA Ratio (Shoot) | -89 | 255 | 91 | 305 | 102.52 | 128 |
| AsA/DHA Ratio (Root) | -45 | 91 | 91 | 128 | 47.5 | 42 |
| **GSH and GSSG Metrics** | | | | | |  |
| GSH (Shoot) | -25 | 35 | 25 | 42 | 5.95 | 5 |
| GSH (Root) | 29 | 52 | 57 | 63 | 25.25 | 26 |
| GSSG (Shoot) | -54 | 5 | 1 | 5 | 59.36 | 63 |
| GSSG (Root) | -24 | 27 | 25 | 26 | 35.8 | 42 |
| GSH/GSSG (Shoot) | 64 | 30 | 25 | 36 | 3.75 | 5 |
| GSH/GSSG (Root) | 69 | 19 | 25 | 30 | -137 | 274 |
| **Soil** | | | | | | |
| Dehydrogenase | -35 | 221 | 184 | 260 | -1.64 | 260 |
| Phosphatase | -38 | 38 | 29 | 50 | 55.98 | 50 |
| Urease | -51 | 258 | 249 | 274 | -135.42 | 274 |
| Catalase | 1 | 46 | 43 | 50 | 69.22 | 50 |
| Amylase | -74 | 180 | 158 | 208 | 53.6 | 208 |
| Sucrase | -6 | -24 | -25 | -19 | -55 | -19 |
| MBC | 110 | 4 | 0.2 | 10 | 4.192 | 10 |
| MBN | 49 | 2 | 2 | 5 | 3.96 | 5 |
| Saturated PLFA | 126 | 0 | -1 | 32 | -1 | 32 |
| G | 76 | 2 | 0.2 | 7 | 2.196 | 7 |
| G- | 124 | 31 | 1 | 55 | 31.69 | 55 |
| Bacteria | 84 | 8 | 0.3 | 17 | 8.27 | 17 |
| Fungi | 81 | 19 | 14 | 56 | 30.34 | 56 |
| Actinomycetes | 152 | 30 | 13 | 39 | 39.1 | 39 |
| Monosaturated PLFA | 131 | 4 | -7 | 13 | -2.72 | 13 |
| Cyclopropyl PLFA | 96 | -2 | -6 | 10 | -8.12 | 10 |
| Precursor PLFA | 149 | 19 | 0.16 | 14 | 19.1296 | 14 |
| G/G- | -22 | -20 | -0.4 | -31 | -20.48 | -31 |
| Fungi/Bacteria | -22 | -20 | -0.4 | -30.7 | -20.48 | -30.7 |
| Saturated PLFA/Monosaturated PLFA | -1 | -3 | 6 | 21 | 3.18 | 21 |
| Cyclopropyl PLFA/Precursor PLFA | -20 | -16 | -6 | -2 | 3.18 | -2 |
| DOC | 49 | 14 | 8 | 13 | -22.96 | -2 |
| NH_4_ | 41 | 13 | 11 | 19 | 20.88 | 13 |
| N0_3_^-^ | -55 | 135 | 143 | 167 | 22.57 | 19 |

**Table S5:** Metabolite concentrations (mean ± SD) categorized as sugars and polyols, amino acids, organic acids, fatty acids, and others from root exudates, determined by GC-MS analysis, across five conditions.

| **Metabolite name** | **Control** | **MMP** | **MP** | **TMP** | **TMMP** |
| --- | --- | --- | --- | --- | --- |
| **Sugars and polyols** | | | | | |
| Glucose | 149.18 ± 26.64 | 199.76 ± 4.77 | 204.09 ± 23.49 | 166.45 ± 3.10 | 243.47 ± 25.81 |
| Glucose-1-P | 6.65 ± 0.48 | 8.25 ± 0.43 | 8.42 ± 0.34 | 11.26 ± 1.10 | 13.42 ± 1.51 |
| Glucose-6-P | 2.97 ± 0.10 | 3.29 ± 0.15 | 3.97 ± 0.22 | 3.54 ± 0.29 | 4.18 ± 0.20 |
| **D-talose** | 25.72 ± 2.85 | 36.38 ± 1.17 | 33.59 ± 3.20 | 34.69 ± 2.49 | 49.42 ± 1.60 |
| Fructose | 534.28 ± 66.65 | 723.11 ± 26.74 | 817.60 ± 86.98 | 659.61 ± 617.09 | 833.44 ± 118.55 |
| Fructose-6-P | 1.46 ± 0.21 | 1.50 ± 0.25 | 1.92 ± 0.12 | 1.70 ± 0.14 | 2.51 ± 0.27 |
| Myo-inositol | 86.47 ± 3.49 | 77.33 ± 3.05 | 100.31 ± 7.46 | 53.90 ± 2.20 | 63.78 ± 4.22 |
| Raffinose | 8.08 ± 0.29 | 14.42 ± 1.86 | 15.10 ± 2.14 | 12.33 ± 2.83 | 15.30 ± 2.97 |
| Ribose | 2.94 ± 0.06 | 3.32 ± 0.36 | 2.47 ± 0.31 | 3.80 ± 0.22 | 4.14 ± 0.30 |
| Trehalose | 1.04 ± 0.46 | 1.89 ± 0.12 | 1.72 ± 0.15 | 0.81 ± 0.02 | 0.63 ± 0.01 |
| Xylitol | 1.08 ± 0.13 | 3.98 ± 0.28 | 2.48 ± 0.10 | 4.43 ± 0.32 | 4.34 ± 0.36 |
| Xylose | 0.70 ± 0.01 | 0.59 ± 0.04 | 0.60 ± 0.02 | 8.80 ± 0.02 | 1.15 ± 0.12 |
| **Melibiose** | 0.99 ± 0.06 | 1.50 ± 0.32 | 1.24 ± 0.14 | 1.00 ± 0.08 | 1.36 ± 0.33 |
| Cellobiose | 0.11 ± 0.03 | 0.14 ± 0.02 | 0.16 ± 0.03 | 0.06 ± 0.00 | 0.15 ± 0.03 |
| Galactinol | 2.79 ± 0.30 | 4.33 ± 0.30 | 4.54 ± 0.21 | 4.93 ± 0.30 | 6.15 ± 0.22 |
| Mannitol | 0.17 ± 0.02 | 0.36 ± 0.04 | 0.31 ± 0.02 | 0.33 ± 0.03 | 0.51 ± 0.03 |
| **Amino acids** | | | | | |
| Alanine | 35.43 ± 2.37 | 64.05 ± 3.62 | 99.62 ± 7.16 | 64.88 ± 3.39 | 119.26 ± 21.21 |
| Aspartic acid | 16.52 ± 1.98 | 41.21 ± 1.72 | 74.21 ± 2.45 | 47.26 ± 1.79 | 135.08 ± 21.39 |
| Glutamic acid | 3.66 ± 0.15 | 7.9 ± 0.13 | 12.96 ± 2.36 | 0.66 ± 0.03 | 19.87 ± 1.66 |
| Glycine | 6.17 ± 0.32 | 8.01 ± 0.11 | 7.33 ± 0.32 | 6.39 ± 0.33 | 8.91 ± 0.37 |
| L-allothreonine | 3.96 ± 0.10 | 15.33 ± 2.86 | 15.37 ± 2.57 | 9.82 ± 0.64 | 22.51 ± 1.61 |
| Oxoproline | 341.00 ± 24.16 | 571.42 ± 22.89 | 606.76 ± 22.04 | 447.63 ± 26.68 | 593.67 ± 18.64 |
| Serine | 4.54 ± 0.40 | 5.32 ± 0.47 | 5.39 ± 0.35 | 7.15 ± 0.18 | 14.93 ± 0.78 |
| Valine | 6.78 ± 0.21 | 21.72 ± 7.17 | 29.34 ± 2.55 | 16.90 ± 1.49 | 35.64 ± 2.44 |
| **Organic acids** | | | | | |
| 3-phosphoglycerate | 1.40 ± 0.24 | 2.19 ± 0.21 | 2.94 ± 0.11 | 1.54 ± 0.21 | 2.97 ± 0.30 |
| 4-hydroxycinnamic acid | 0.26 ± 0.02 | 0.34 ± 0.04 | 0.30 ± 0.03 | 0.41 ± 0.02 | 0.41 ± 0.04 |
| Benzoic acid | 0.92 ± 0.06 | 1.02 ± 0.08 | 1.14 ± 0.08 | 1.03 ± 0.11 | 1.02 ± 0.10 |
| Citric acid | 104.89 ± 13.61 | 140.86 ± 35.29 | 161.82 ± 20.13 | 180.75 ± 11.56 | 133.13 ± 21.97 |
| Dehydroshikimic acid | 1.03 ± 0.08 | 0.52 ± 0.02 | 0.53 ± 0.04 | 2.54 ± 0.32 | 2.39 ± 0.25 |
| D-glyceric acid | 10.10 ± 0.32 | 10.99 ± 1.30 | 14.91 ± 3.45 | 15.09 ± 2.67 | 14.78 ± 2.79 |
| Ferulic acid | 0.38 ± 0.03 | 0.71 ± 0.04 | 0.61 ± 0.03 | 0.75 ± 0.02 | 0.94 ± 0.04 |
| L-malic acid | 101.13 ± 7.03 | 106.51 ± 13.15 | 123.55 ± 29.26 | 155.25 ± 13.09 | 140.86 ± 17.42 |
| Oxalic acid | 523.08 ± 25.26 | 418.05 ± 29.15 | 420.80 ± 44.04 | 438.52 ± 25.50 | 261.66 ± 20.69 |
| pyruvic acid | 1.29 ± 0.17 | 1.98 ± 0.09 | 1.59 ± 0.25 | 2.60 ± 0.23 | 3.52 ± 0.27 |
| Quinic acid | 35.72 ± 3.20 | 25.16 ± 3.32 | 42.81 ± 3.74 | 95.23 ± 2.74 | 53.63 ± 3.00 |
| Salicylic acid | 3.86 ± 0.29 | 6.95 ± 0.33 | 7.21 ± 0.28 | 6.09 ± 0.21 | 6.07 ± 0.30 |
| Succinic acid | 20.58 ± 1.79 | 21.15 ± 1.79 | 23.43 ± 3.32 | 34.39 ± 2.32 | 24.12 ± 2.78 |
| **Fatty acids** | | | | | |
| Linoleic acid | 0.51 ± 0.03 | 0.51 ± 0.02 | 0.44 ± 0.04 | 0.65 ± 0.02 | 0.61 ± 0.02 |
| Linolenic acid | 1.12 ± 0.21 | 1.16 ± 0.06 | 1.12 ± 0.14 | 1.70 ± 0.08 | 1.44 ± 0.20 |
| Palmitic acid | 147.68 ± 5.16 | 149.14 ± 1.65 | 133.94 ± 4.04 | 130.53 ± 4.33 | 147.70 ± 19.77 |
| Stearic acid | 96.12 ± 2.62 | 93.03 ± 3.82 | 87.02 ± 1.93 | 81.36 ± 2.60 | 124.81 ± 20.53 |
| **Others** | | | | | |
| Ethanolamine | 100.38 ± 11.61 | 107.42 ± 16.60 | 117.40 ± 14.70 | 111.31 ± 11.63 | 142.65 ± 6.27 |
| Methyl Phosphate | 28.90 ± 0.89 | 42.97 ± 2.83 | 40.87 ± 3.46 | 79.32 ± 3.13 | 83.34 ± 3.33 |
| Phosphate | 1873.19 ± 32.64 | 2197.34 ± 55.89 | 2036.57 ± 86.09 | 2183.52 ± 43.13 | 535.08 ± 35.30 |

**Table S6:** Log2 fold changes in metabolite concentrations (mean ± SD) in root exudates, determined by GC-MS. The MP treatment is compared with the untreated control, while TDMPs, METMPs and TDMETMPs are compared with the treated control (MPs stress).

| **Metabolite name** | **MP** | **TDMP** | **METM** | **TDMETMP** |
| --- | --- | --- | --- | --- |
|  | Fold changes log2 | | | |
| **Sugars and polyols** | | | | |
| Glucose | 0.45 ** | 0.71 ** | 0.16 | 0.25 * |
| Cellobiose | 0.48 * | 0.46 * | 0.79 * * | 0.02 |
| D-talose | 0.38 * * | 0.94 * * | 0.43 * * | 0.56 * * |
| Fructose | 0.49 * * | 0.81 * * | 0.26 * * | 0.32 * * |
| Fructose-6-P | 0.40 * * | 0.78 * * | 0.22 * | 0.38 * * |
| Galactinol | 0.70 * * | 1.14 * * | 0.82 * * | 0.44 * * |
| Glucose-1-P | 0.34 * * | 1.01 * * | 0.76 * * | 0.67 * * |
| Glucose-6-P | 0.42 * * | 0.49 * * | 0.25 * * | 0.07 |
| Mannitol | 0.86 * * | 1.57 * * | 0.93 * * | 0.70 * * |
| Melibiose | 0.33 * * | 0.47 * | 0.02 | 0.13 |
| Myo-inositol | 0.21 * * | -0.44 * * | -0.68 * * | -0.65 * * |
| Raffinose | 0.90 * * | 0.92 * * | 0.61 * * | 0.02 |
| Ribose | -0.25 * * | 0.49 * * | 0.37 * * | 0.74 * * |
| Trehalose | 0.72 * * | -0.73 | -0.37 | -1.45 * * |
| Xylitol | 1.20 * * | 2.01 * * | 2.04 * * | 0.81 * * |
| Xylose | -0.22 * * | 0.72 * * | 0.33 * * | 0.94 * * |
| **Amino acids** | | | | |
| Alanine | 1.49 * * | 1.75 * * | 0.87 * * | 0.26 |
| Aspartic acid | 2.17 * * | 3.03 * * | 1.52 * * | 0.86 * * |
| Glutamic acid | 1.82 * * | 2.44 * * | -2.48 * * | 0.62 * * |
| Glycine | 0.25 * * | 0.53 * * | 0.05 | 0.28 * * |
| L-allothreonine | 1.96 * * | 2.51 * * | 1.31 * * | 0.55 * * |
| Oxoproline | 0.83 * * | 0.80 * * | 0.39 * * | -0.03 |
| Serine | 0.25 * * | 1.72 * * | 0.65 * * | 1.47 * * |
| Valine | 2.11 * * | 2.39 * * | 1.32 * * | 0.28 * * |
| **Organic acids** | | | | |
| Oxalic acid | -0.31 * * | -1.00 * * | -0.25 * * | -0.69 * * |
| 3-phosphoglycerate | 1.08 * * | 1.09 * * | 0.14 | 0.01 |
| 4-hydroxycinnamic acid | 0.24 * | 0.69 * * | 0.67 * * | 0.45 * * |
| Benzoic acid | 0.31 * * | 0.16 | 0.18 * | -0.15 |
| Citric acid | 0.63 * * | 0.34 * | 0.78 * * | -0.28 * |
| Dehydroshikimic acid | -0.96 * * | 1.21 * * | 1.30 * * | 2.17 * * |
| D-glyceric acid | 0.56 * * | 0.55 * * | 0.58 * * | -0.01 |
| Ferulic acid | 0.69 * * | 1.31 * * | 0.97 * * | 0.62 * * |
| L-malic acid | 0.29 | 0.48 * * | 0.62 * * | 0.19 |
| Pyruvic acid | 0.29 * | 1.45 * * | 1.00 * * | 1.15 * * |
| Quinic acid | 0.26 * * | 0.59 * * | 1.41 * * | 0.33 * * |
| Salicylic acid | 0.90 * | -0.19 * * | 0.66 * | -0.25 * * |
| Succinic acid | 0.19 | 0.23 * | 0.74 * * | 0.04 |
| **Fatty acids** | | | | |
| Linolenic acid | 0.01 | 0.37 * * | 0.60 * * | 0.36 * * |
| Linoleic acid | -0.23 * * | 0.25 * * | 0.34 * * | 0.48 * * |
| Palmitic acid | -0.14 * * | 0 | -0.18 * * | 0.14 |
| Stearic acid | -0.14 * * | -0.19 * * | -0.24 * * | 0.52 * * |
| **Others** | | | | |
| Eethanolamine | 0.23 | 0.51 * * | 0.15 | 0.28 * * |
| Methyl Phosphate | 0.50 * * | 1.53 * * | 1.46 * * | 1.03 * * |
| Phosphate | 0.12 * * | -1.81 * * | 0.22 * * | -1.93 * * |

**Table S7:** Structural equation modeling (SEM) fit statistics for plant responses under four treatments: MP stress (MP vs. Control), TDMP (TDMP vs. MP), METMP (METMP vs. MP), and TDMETMP (TDMETMP vs. MP). Columns include degrees of freedom (DoF), baseline DoF, chi-squared (chi2), chi-squared p-value, baseline chi-squared, comparative fit index (CFI), goodness-of-fit index (GFI), adjusted goodness-of-fit index (AGFI), normed fit index (NFI), Tucker-Lewis index (TLI), root mean square error of approximation (RMSEA), Akaike information criterion (AIC), Bayesian information criterion (BIC), and log-likelihood (LogLik), derived from data encompassing ROS-scavenging enzymes, plant growth traits, oxidative stress markers, antioxidant enzymes, and metabolite diversity.

| **Treatments** | **DoF** | **DoF Baseline** | **chi2** | **chi2 p-value** | **chi2 Baseline** | **CFI** | **GFI** | **AGFI** | **NFI** | **TLI** | **RMSEA** | **AIC** | **BIC** | **LogLik** |
| --- | --- | --- | --- | --- | --- | --- | --- | --- | --- | --- | --- | --- | --- | --- |
| **MPs stress** | 1258 | 1326 | 337.9073206 | 1 | 1281.987042 | -19.90504053 | 0.736419083 | 0.722171465 | 0.736419083 | -21.03504273 | 0 | 127.3642265 | 102.3753628 | 56.31788677 |
| **TDMPs** | 1258 | 1326 | 439.9357241 | 1 | 1275.722327 | -15.27092565 | 0.655147743 | 0.63650708 | 0.655147743 | -16.15043514 | 0 | 93.35475863 | 68.36589493 | 73.32262069 |
| **METMPs** | 1258 | 1326 | 473.8754522 | 1 | 1269.15623 | -12.79437983 | 0.626621656 | 0.606439042 | 0.626621656 | -13.54002198 | 0 | 82.04151595 | 57.05265226 | 78.97924203 |
| **TDMETMPs** | 1258 | 1326 | 345.9196419 | 1 | 1284.762513 | -21.11774819 | 0.730752074 | 0.716198132 | 0.730752074 | -22.31330215 | 0 | 124.6934527 | 99.70458901 | 57.65327365 |
